# *Baat* Gene Knockout Alters Post-Natal Development, the Gut Microbiome, and Reveals Unusual Bile Acids in Mice

**DOI:** 10.1016/j.jlr.2022.100297

**Published:** 2022-10-13

**Authors:** Kerri A. Neugebauer, Maxwell Okros, Douglas V. Guzior, Jeremiah Feiner, Nicholas J. Chargo, Madison Rzepka, Anthony L. Schilmiller, Sandra O’Reilly, A. Daniel Jones, Victoria E. Watson, James P. Luyendyk, Laura R. McCabe, Robert A. Quinn

**Affiliations:** 1 Department of Biochemistry and Molecular Biology, Michigan State University, East Lansing, MI, USA; 2 Department of Microbiology and Molecular Genetics, Michigan State University, East Lansing, MI, USA; 3 Department of Physiology, Michigan State University, East Lansing, MI, USA; 4 Mass Spectrometry and Metabolomics Core, Michigan State University, East Lansing, MI, USA; 5 Department of Pathobiology and Diagnostic Investigation, College of Veterinary Medicine, Michigan State University, East Lansing, MI, USA; 6 Department of Pharmacology and Toxicology, Michigan State University, East Lansing, MI, USA

**Keywords:** steroid detergents, microbiome interactions, taurocholic acid, conjugation, bile acid-CoA:amino acid N-acyltransferase, peroxisomal acyltransferases, fat absorption, liver, cysteamine, taurine, ASVs, amplified sequence variants, BAAT, bile acid-CoA:amino acid N-acyltransferase, BAs, bile acids, CA, cholic acid, CDCA, chenodeoxycholic acid, Cys-CA, cysteinocholic acid, GCA, glycocholic acid, MCBA, microbially conjugated bile acid, MMRRC, Mutant Mouse Resource and Research Center, MWU, Mann-Whitney U test, PASH, periodic-acid Schiff, PCoA, principal co-ordinate analysis, PERMANOVA, permutational multivariate analysis of variance, RF, random forest, TCA, taurocholic acid, TmCA, tauromuricholic acid

## Abstract

Bile acids (BAs) are steroid detergents in bile that contribute to fat absorption, cell signaling, and microbiome interactions. The final step in their synthesis is amino acid conjugation with either glycine or taurine in the liver by the enzyme bile acid-CoA:amino acid N-acyltransferase (BAAT). Here, we describe the microbial, chemical, and physiological consequences of *Baat* gene knockout. *Baat*^*-/-*^ mice were underweight after weaning but quickly exhibited catch-up growth. At three weeks of age, KO animals had increased phospholipid excretion and decreased subcutaneous fat pad mass, liver mass, glycogen staining in hepatocytes, and hepatic vitamin A stores, but these were less marked in adulthood. Additionally, KO mice had an altered microbiome in early life. Their BA pool was highly enriched in cholic acid but not completely devoid of conjugated BAs. KO animals had 27-fold lower taurine-conjugated BAs than wild type in their liver but similar concentrations of glycine-conjugated BAs and higher microbially conjugated BAs. Furthermore, the BA pool in *Baat*^*-/-*^ was enriched in a variety of unusual BAs that were putatively sourced from cysteamine conjugation with subsequent oxidation and methylation of the sulfur group mimicking taurine. Antibiotic treatment of KO mice indicated the microbiome was not the likely source of the unusual conjugations, instead, the unique BAs in KO animals were likely derived from the peroxisomal acyltransferases *Acnat1* and *Acnat2*, which are duplications of *Baat* in the mouse genome that are inactivated in humans. This study demonstrates that BA conjugation is important for early life development of mice.

Bile acids (BAs) are physiological detergents synthesized in the liver from cholesterol and are responsible for fat solubilization in the gut. As the final step in BA synthesis, bile acid-CoA:amino acid N-acyltransferase (BAAT) performs an acyl-conjugation of cholic acid (CA) or chenodeoxycholic acid (CDCA) with either the amino acids glycine or taurine (1). Taurine- and glycine-bound CA derivatives, taurocholic acid (TCA) and glycocholic acid (GCA), were first isolated and characterized by Adolf Strecker in 1848 and their structures were first proposed in 1927, for which Heinrich Otto Weiland was awarded the Nobel Prize (2). The *BAAT* gene is expressed in the liver and its acyl-conjugation reaction with either glycine or taurine creates the four primary conjugated BAs that are transported to the gallbladder and stored until needed for fat digestion after a meal. BAAT has high specificity for these two amino acids (Human BAAT: taurine *K*_m_=1.1 mM, glycine *K*_m_=5.8 mM) (3, 4), which is somewhat surprising considering the diversity of lipids in the mammalian diet needing to be solubilized and the potential for other amino acid conjugates to expand solubilization capacity. Nevertheless, TCA and GCA are the principal conjugates found in mammals, with other examples initially thought to be extremely rare (3, 5). However, a recent study identified the microbiota as a significant source of BA conjugation (6). In fact, a wide variety of gut bacteria are able to conjugate BAs with other amino acids, but the importance and impact of this biochemistry remains unknown (7).

A primary function of conjugated BAs is to solubilize dietary fat (2). These molecules are amphipathic along their planar axis, enabling the formation of micelles with polar fatty acids and other dietary lipids facilitating their absorption in the ileum (8, 9). As such, conjugated BAs are believed to promote fat soluble vitamin absorption, another important function of the BAAT enzyme. Humans with mutations in the gene coding for the BAAT enzyme or BA-coenzyme A ligase (*SLC27A5*) show reduced absorption of fat soluble vitamins A, K, D, and E (10). Another proposed function of BAs is shaping the structure of the microbiome because they are believed to be inherently antimicrobial. Despite this widely accepted nature of BAs, direct in vitro demonstration of antimicrobial activity has been varied depending on the microorganism (11, 12, 13). The inhibitory effect of conjugated BAs may also occur through signaling of the farnesoid-X receptor and the subsequent production of antimicrobial compounds (8, 14). Considering the concentration of conjugated BAs in the upper gastrointestinal (GI) tract (as high as 10 mM after a meal (8)), there is a need to better understand how BAAT function affects gut physiology and microbiology. Though reports of humans with defects in BAAT are available (10, 15), providing some insight into the physiological consequences of BA conjugation, little is known about the effects of BA conjugation on host physiology.

Here, we comprehensively profile the biochemistry, microbiology, and physiology of mice with a C-terminal deletion in *Baat* (*Baat*^*-/-*^, KO). Using untargeted and targeted metabolomics and 16S rRNA gene microbiome sequencing, we show that *Baat*^*-/-*^ mice have significantly disrupted BA profiles including unique acyl-conjugates with other sulfur-containing residues. *Baat*^*-/-*^ mice have greatly reduced TCA but are not devoid of taurine- or glycine-conjugated BAs. The knockouts (KOs) were significantly disrupted early in life, but most physiological measures, including microbiome profiles, were not significantly altered into adulthood. This study shows that BAAT may not be the only BA-conjugating enzyme in mice and that unique acyl-conjugations in these animals can substitute for the loss of taurine conjugation.

## Materials and Methods

### Mice

Cryo-preserved heterozygous sperm for strain C57BL/6NCrl-*Baat*^*em1(IMPC)Mbp*^/Mmucd (RRID:MMRRC_043495-UCD), originally created by Cas9 editing according to (16, 17), was obtained from the Mutant Mouse Resource and Research Centers (MMRRC, University of California Davis). The genetic background of the animals is described on the MMRRC website (https://www.mmrrc.org/catalog/sds.php?mmrrc_id=43495). Exon 2 ENSMUSE00000229562 and flanking splicing regions were constitutively deleted from the *Baat* gene ENSMUST00000043056.8 using CRISPR Cas9 gene editing technology in mouse zygotes (16, 17); this deletion includes the highly conserved ^328^Asp and ^362^His in the active site (18). Cryo-recovery was performed via in-vitro fertilization at the Michigan State University Transgenic and Genome Editing Facility. The resulting heterozygotes were combined in a duo breeding scheme to obtain heterozygous (HET), wildtype (WT), and KO genotypes. The genotyping probes and assay were developed and executed by the University of California Davis MMRRC and Transnetyx, Inc. (Cordova, TN, see supplemental methods). All further husbandy was performed using duo or trio breeding groups in the following combinations: HET by HET, male KO by female HET, or KO by KO. Mice were housed at a maximum of five per cage, fed sterilized Teklad 8940 chow (Teklad, Madison, WI) *ad libitum,* and were maintained on a 12-h light/dark cycle. All animal procedures were approved by the Michigan State University Institutional Animal Care and Use Committee and conformed to NIH guidelines. Animal numbers per sex, genotype, and experiment are shown in supplemental Table S1. Further physiological characterization of *Baat*^*-/-*^ animals was initially performed by the source laboratory (16, 17) and is available online at: https://www.mousephenotype.org/data/genes/MGI:106642#phenotypes-section.

### Sample collection

Animals used for growth measurements and fecal sample collection through the first 8 weeks of life included 14 KO females, 17 KO males, 10 WT females, and WT KO males. In addition, fecal samples from 24 HET females and 24 HET males were also used for fecal sample analysis, but data from these animals were not included elsewhere. Four animals died or were euthanized before adulthood. Longitudinal fecal samples (HET, KO, and WT) were collected weekly starting at three weeks through eight weeks by temporarily placing the mice in clean plastic cups. Pellets were immediately frozen and stored at −80°C. For tissue collection, animals were sacrificed using anesthesia (isoflurane inhalation) at either 3- or 8-weeks of age including male (n = 7–8 per genotype) and females (n = 7–8 per genotype), and blood was collected for serum via cardiac puncture. Following physical decapitation, the brain, kidneys, liver, gallbladder, ileum, duodenum, jejunum, cecum, and colon were dissected from each mouse, flash frozen in liquid nitrogen, and stored at −80°C. The gallbladder was collected with care such that it did not burst, and all contents were contained within the sample for analysis. DNA, RNA, and metabolites were analyzed after splitting the samples for different processing workflows. For DNA and metabolites, phosphate-buffered saline was added (3:1, weight:volume) to the fecal samples and homogenized using a Bead Ruptor® 96 at 20 Hz (Omni International, Inc, Kennesaw, GA). For RNA, samples were homogenized (3:1 weight:volume) in DNA/RNA Shield® (Zymo® Research Inc).

### Histopathologic examination and physiological measurements

After initial sampling for omics data analysis, liver, ileum, and distal and proximal colon were sampled from 3-week-old male animals for histological analysis and physiology measurements (n = 4 KO and n = 5 WT). One female WT and one female KO mouse were also sampled at 8-weeks but initial histological analysis showed little differences between genotypes, hence the focus on 3-week-old animals. Tissues were thawed and then fixed in 10% neutral buffered formalin. Sections of each tissue were paraffin embedded, sectioned at 5 μm thickness, and stained with hematoxylin and eosin or periodic-acid Schiff (PASH) reaction for the 3-week-old mice and hematoxylin and eosin only for the 8-week-old mice. Tissues were microscopically examined by a board-certified veterinary anatomic pathologist. Sections of intestine were analyzed for number of goblet cells, number of Paneth cells, villus to crypt ratio or mucosal depth depending on section, and presence of luminal bacteria. In the liver, the degree of hepatocellular vacuolation was determined as mild, moderate, or high.

### 16s rRNA gene sequencing

DNA was extracted with a DNeasy® PowerSoil® Kit according to the manufacturer's instructions. To ensure the DNA quality before sequencing, the 16s rRNA gene region was PCR amplified with GoTaq Green Master Mix, 16S rRNA gene primers 27F (AGAGTTTGATCMTGGCTCAG), and 1492R (TACGGYTACCTTGTTACGACTT) under the following conditions: 95°C for 5 min; 30 cycles of 95°C for 1 min, 48°C for 30 s, and 72°C for 2 min; then 72°C for 10 min.

Samples were submitted to the Michigan State Research Technology Support Facility Genomics Core for 16S rRNA gene-V4 amplicon library preparation and sequencing. The V4 hypervariable region of the 16S rRNA gene was amplified using Illumina compatible, dual-indexed primers 515F/806R following the protocol developed in (19). PCR products were batch normalized using an Invitrogen® SequalPrep DNA Normalization plate and product recovered from the pooled plates. The pool was concentrated using a QIAquick® Spin column and AMPure XP® magnetic beads. The quality of the pool was evaluated using a combination of Qubit dsDNA HS, Agilent 4200 TapeStation HS DNA1000, and Invitrogen Collibri Illumina Library Quantification qPCR assays. The pool was then loaded onto one MiSeq v2 Standard flow cell and sequencing was carried out in a 2x250 bp paired end format using a MiSeq v2 500 cycle reagent cartridge. Custom sequencing and index primers complementary to the 515F/806R oligomers were added to appropriate wells of the reagent cartridge. Base calling was done by Illumina Real Time Analysis v1.18.54 and output of real time analysis was demultiplexed and converted to FastQ format with Illumina Bcl2fastq v2.20.0. The resulting FASTA files were analyzed using Qiita v2019.09 93f90ef (20).

### Metabolomics

Homogenates of tissue and feces were extracted by the addition of a 3:1 volumes of ice cold 70% methanol, vortexed for 30 s, and then extracted for 2 h at room temperature. Serum samples were extracted without homogenization and run and analyzed separately using liquid chromatography with tandem mass spectrometry (LC-MS/MS) due to the dilution effects of the serum on retention time indices. The cold methanol was spun at 10,000 *g* for 1 min to precipitate protein and the extract was used for mass spectrometry analysis. Blank extractions containing only the extraction microcentrifuge tubes were used throughout the LC-MS/MS method to identify background contaminants for removal. Methanol extracts were diluted 1:1 (volume:volume) in 96-well plates with 50% methanol containing 2.5 μg/ml phenolsulfonphthalein as an internal standard and analyzed on a Thermo™ QExactive™ mass spectrometer coupled to a Vanquish Ultra-High-Performance Liquid Chromatography system (ThermoFisher). The mobile phase was 0.1% formic acid in Milli-Q water (channel A) and acetonitrile containing 0.1% formic acid (channel B). LC separation was performed on a reverse phase Waters® Acquity® UPLC BEH C-18 column, 2.1 mm × 100 mm. The chromatographic runs were 12 minlong with linear gradients as follows: 0–1 min 2% B and 1–8 min 2%–100% B. This 100% B solution was then held for 2 min followed by a switch to 2% B for the remaining 2 min. The injection volume was 10 μl, the flow rate 0.40 ml/min, and the column temperature was 60°C. Positive mode electrospray ionization was used, and mass spectra were obtained using a data-dependent analysis method with full MS^1^ survey scans (35,000 resolution, *m/z* 100–1500) followed by MS^2^ spectra collected for five precursor ions per survey scan (17,500 resolution, fixed first mass of *m/z* 50). Pseudo MS^3^ spectra were obtained with a slightly modified data-dependent analysis method using an in-source collision-induced dissociation setting of 70.

Raw files (.raw) were converted to the .mzXML format for processing via GNPS Vendor Conversion (UCSD, San Diego, CA) and analyzed with MZmine 2.53 software and GNPS (21, 22). The different sample collections were run and processed in separate batches as follows: 3-week-old mice tissue batch, 8-week-old mice tissue batch, fecal sample batch, and antibiotic experiment batch. Serum samples from both age groups were processed separately through MZmine as it became apparent during the run that the dilution effect of the serum during extraction altered the chromatographic retention times. MZmine parameters were adjusted based on the peak shape and behavior of each batch samples set, but core parameters were as follows: MS^1^ noise level (variable), MS^2^ noise level 2000, m/z tolerance 0.02 or 7 ppm, chromatogram retention time tolerance 0.1 min, maximum charge isotopes 3, and only MS^1^ peaks with MS^2^ spectra were maintained after alignment. The abundance of each feature in the feature table was normalized to the total ion current prior to any downstream analysis. Feature-based molecular networking for all samples was performed with a parent and fragment mass ion tolerance of 0.02 Da, a cosine score of 0.65, and a minimum matched peaks of 4. Library searching used a cosine score of 0.7. Feature-based molecular networking job links for each dataset are available in the supplemental Table S6. The resulting molecular network was imported to Cytoscape 3.8.0 (https://cytoscape.org/) for further analysis. Raw mass spectrometry data is publicly available at massive.ucsd.edu under ID MSV000088761.

### Gene expression analysis

RNA was isolated from mouse tissue after homogenization in DNA/RNA Shield Stabilization Solution (Zymo, Irvine, CA) using Quick-RNA Miniprep Plus kit (Zymo) according to the manufacturer’s instructions. The resulting RNA was checked for quality using a Nanodrop. cDNA was synthesized by reverse transcription-PCR using SuperScript II Reverse Transcriptase Kit and oligo dT primers (Invitrogen, Carlsbad, CA). Real time PCR was carried out as follows: 50°C for 2 min, 95°C for 10 min, then 40 cycles of 95°C for 15 s and 60°C for 1 min with SYBR Green PCR Master Mix (Applied Biosystems, Carlsbad, CA). Gene expression was evaluated using the ΔΔCT method normalizing to the housekeeping gene *m36b4* (F: 5′-ACCTCCTTCTTCTTCCAGGCTTT-3′, R: 5′-CCCACCTTGTCTCCAGTCTTT-3′) as described in (6). Genes evaluated are as follows: BAATF – 5′-TGTGATGAATAGCCCCTACCA-3′; BAATR – 5′-AGGACTGACGACTATGTCTTGTA-3′ (23), ACNAT1F - 5′-GAGGCAGCAACTGTGGTGACT-3'; ACNAT2R - 5′-TGAGACTGTATGTTTTCCTTGCTCTAC-3', ACNAT2F - 5′-AAGCGGGAACAGATTCAAGAAG-3′; ACNAT2R - 5′-ACGAAATTCAACTAGACCCCCA-3′ (24).

### Bacterial strain, growth media, and chemicals

*Enterocloster bolteae* BAA-613 (ATCC), the type-strain, was the sole strain used and each experiment was repeated in triplicate. All experiments were performed in a COY anaerobic chamber (Coy) containing at 98% nitrogen and 2% hydrogen atmosphere. Overnight cultures of *E. bolteae* were started from −80°C stocks grown in Reinforced Clostridial Medium (RCM, Merck), prepared according to the manufacturer’s instructions. Following overnight culture, optical density was measured at 600 nm. Cultures were then diluted to a final *A*_600_ of 0.01 in 1.5 ml microcentrifuge tubes with or without 1 mMCA (Sigma) and 0.1 mM taurine (Thermo Scientific) to a final volume of 1 ml. These were then incubated anaerobically for 16 h at 37°C. Final *A*_600_ was measured via spectrophotometry. Culture extractions were performed by adding ice cold methanol to whole bacterial cell culture at a final concentration of 60% methanol followed by overnight incubation at 4°C. Extracts were stored at −80°C prior to mass spectrometry analysis. Samples were centrifuged at 10,000 rpm for 5 min to pellet cell debris followed by dilution 1:1 (v:v) in 50% methanol in glass vials. Mass spectrometry data generation and analyses were performed as described above with attention to the feature abundance of TCA.

### Microbiota depletion experiment

Fecal samples were collected, and weight was recorded twice a week for 4-week-old KO (n = 5) and WT mice (n = 5) for two weeks to establish a baseline. At six weeks, five antibiotics (0.5 mg/ml ampicillin, 0.5 mg/ml neomycin, 0.5 mg/ml metronidazole, 0.5 mg/ml gentamicin, and 0.25 mg/ml vancomycin) and 4 mg/ml sucralose was added to drinking water for two weeks (25). The control group only received 4 mg/ml sucralose. The water was changed every three days to maintain activity of the antibiotics. The amount of water drank for each cage was monitored. The mice were weighed, and fecal samples were collected twice a week while on antibiotics/sucralose or sucralose. After two weeks on antibiotics/sucralose or sucralose, the mice were humanely sacrificed using isoflurane and physical decapitation and necropsied for blood, gallbladder, liver, cecum, and ileum collection. Sample sizes for this experiment are available in supplemental Table S1.

### Statistical analysis

Weights of mice were compared at each timepoint within genotypes using the Mann-Whitney *U* test (MWU-test). Beta-diversity of the metabolome data was quantified using Bray-Curtis distance and UniFrac (26) distance for the microbiome. The beta-diversity variation was visualized in principle coordinate analysis (PCoA) space using EMPeror (27). Permutational multivariate analysis of variance (PERMANOVA) testing was used to determine if there were significant differences between genotypes across the entire metabolome and/or microbiome data. Bray-Curtis and UniFrac differences across genotypes were also compared within genotypes by calculating the distances between pairwise samples based on genotype classification. These differences in pairwise beta-diversity were then tested for significance with the MWU-test. The same across/within genotype beta-diversity comparisons were also done for the fecal metabolome and tested for significance with the MWU-test. All other metabolite or microbiome comparisons between genotypes were tested with the MWU-test at an alpha-level of 0.05.

## Results

### Growth and pathology of *Baat*^*-/-*^ versus WT

**Fig. 1:**
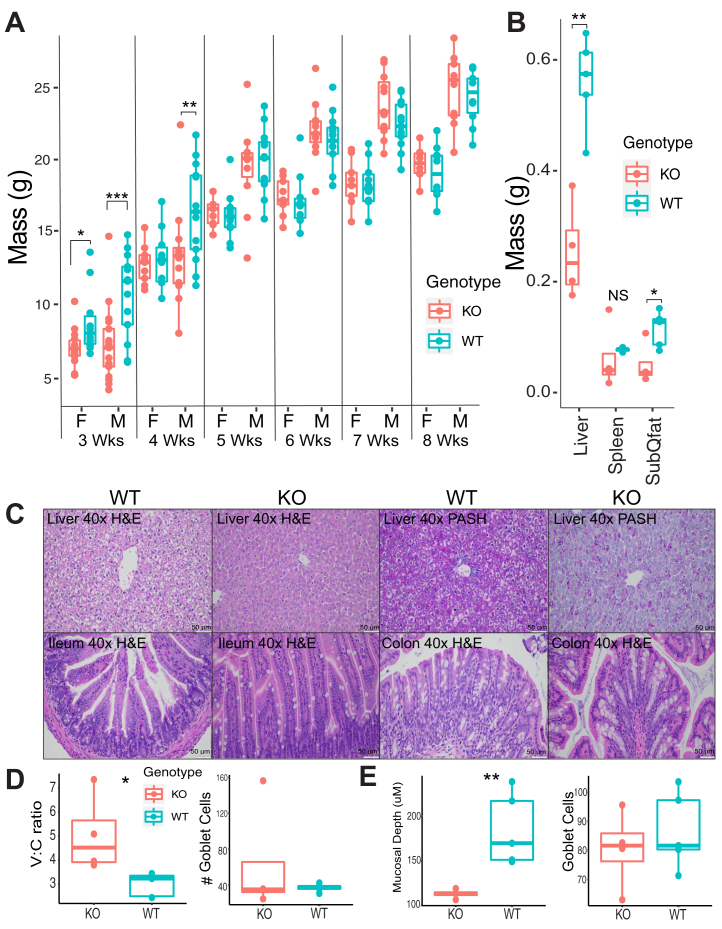
Growth profile and histopathology of *Baat*^*-/-*^ versus WT. A: Total body mass growth profile of *Baat*^*-/-*^ mice from 3- to 8-weeks by sex and genotype (14 KO males, 9 KO females, 13 WT males, and 10 WT females). Significance was tested with the MWU-test at each time point for each gender. B: Organ and subcutaneous (SubQ) fat pad masses in male WT (n = 5) and KO mice (n = 4) at 3-weeks of age. C: Histological images of KO and WT mice liver (top row), ileum (bottom row, left 2 panels), and colon (bottom row, right 2 panels) at 3-weeks of age. Slides were stained with either hematoxylin/eosin (H & E) or Periodic acid-Schiff-Hematoxylin (PASH) for the liver and visualized at 40x magnification as labeled. D: Villus:Crypt ratio and Goblet cells counts in the ileum of male mice by genotype. E: mucosal depth and Goblet cell counts in the colon of male mice by genotype (n = 5 (WT) and 4 (KO) per group) (unpaired *t*-test). ∗*P* < 0.05, ∗∗*P* < 0.01, ∗∗∗*P* < 0.001. MWU, Mann-Whitney U test.

Cryopreserved sperm from mouse strain C57BL/6NCrl-*Baat*^*em1(IMPC)Mbp*^/Mmucd (*Baat*^*-/-*^) was artificially inseminated into a C57BL/6 female and pups were born and genotyped as *Baat*^*+/-*^ (HET, genotyping protocol in supplemental data). This mouse strain has a deletion in exon 2 and a section of the following intron of *Baat* resulting in a frame shift and predicted premature stop codon truncating the C-terminus (see supplementary data). HET pups were then bred in a second generation to produce *Baat*^*-/-*^ animals. Subsequent breeding rounds showed that KO females were able to successfully breed with KO male mice, but these breeding pairs had reduced fecundity. Out of five initial breeding pairs, only one produced a litter where all pups survived, one litter was partial, and three of these litters contained all stillborn pups. Thus, HET crosses of *Baat*^*+/-*^ female by *Baat*^*-/-*^ male breeding was used as the primary means of producing *Baat*^*-/-*^ pups followed by genotyping of each pup for experiments. *Baat*^*-/-*^ female and male pups were underweight after weaning as compared to WT littermates at 3-weeks (Fig. 1A). Only male mice remained significantly underweight at 4-weeks. Both sexes quickly exhibited catch-up growth such that they were no longer statistically different from WT (*Baat*^*+/+*^, WT) after 4 weeks and remained so into adulthood. Grossly, 8-week-old KO animals showed no signs of significant pathology, difference in organ size/color, or behavioral alterations (supplemental Fig. S1). The liver mass and subcutaneous fat pad mass of male mice at 3 weeks of age were significantly lower in KO mice than WT but the spleen mass was not (Fig. 1B). Length of the GI tract from duodenum to anus was not significantly different between 3-week-old KO compared to WT mice (n=5 per group, *P* = 0.34, Student’s *t*-test).

Microscopic examination of livers and small intestine sections from a subset of WT and KO mice at 3- and 8-weeks was performed. Consistent with similar body and organ weights at 8-weeks of age, no discernible differences were noted in liver and ileum (supplemental Fig. S2). Therefore, we focused histological analyses on KO and WT animals at 3-weeks of age. In the WT mice, hepatocytes variably contained indistinct cytoplasmic vacuoles and had central nuclei, indicating glycogen storage (Fig. 1C). PASH-stained sections similarly showed predominantly diffuse PASH staining in the hepatocytes (Fig. 1C) of WT mice. However, KO mice hepatocytes contained fewer vacuoles and all KO mice had decreased PASH staining indicating a decrease in glycogen storage (Fisher’s exact test *P* = 0.0005). Two livers of KO mice contained increased numbers of Kupffer cells within sinusoids occasionally forming small aggregates. Examination of the intestine indicated significant differences in villus to crypt ratios, in the sections of ileum and mucosal depth in the colon (Fig. 1D, E). However, the mucosal structure was not significantly different. Further histological analysis in the GI tract of these animals, particularly comparing pups to adults and with other staining methods, may reveal more abnormalities in the absorptive tissue of KO mice. Other physiological parameters compared between *Baat* KO and WT were initially performed by the animal’s source institution as part of the International Mouse Phenotyping Consortium (16, 17) and are available online (see Materials and Methods).

### Tissue and fecal metabolome variation in KO and WT mice

**Fig. 2:**
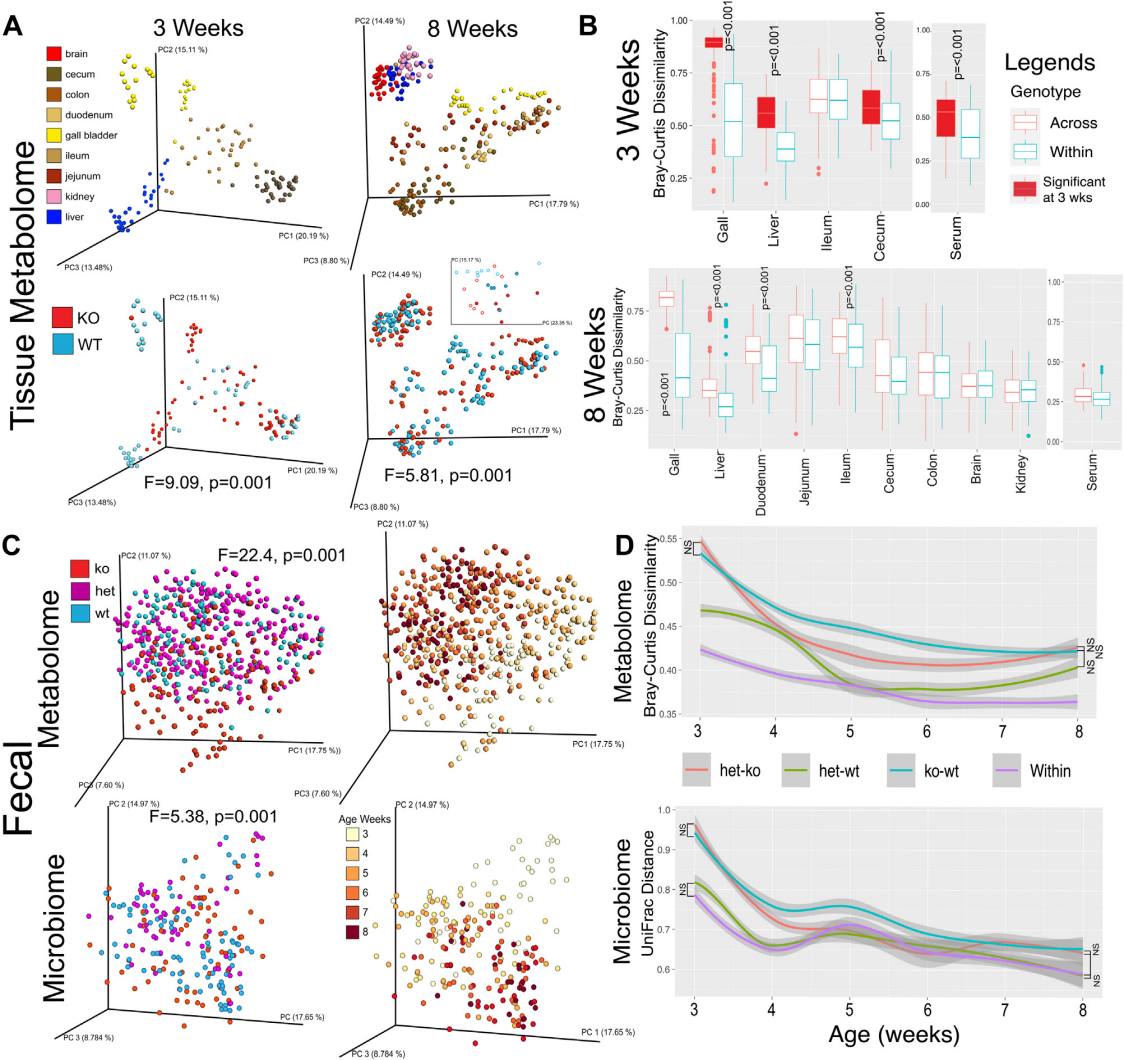
Metabolome and microbiome variation in *Baat*^*-/-*^, *Baat*^*+/-*^ versus *Baat*^*+/+*^. A: PCoA plots generated on metabolomic data from tissue samples after calculation of pairwise Bray-Curtis distances of each sample. Data was analyzed and plotted separately for mice aged 3- and 8-weeks, and the plots are colored by tissue type and genotype separately. An inset 2-D PCoA plot of the serum metabolome is also shown with the same color scheme (circles = male, balls = females). The F-value and *P*-value calculated from the PERMANOVA test is shown for the genotype category. B: Boxplots of the Bray-Curtis distances of the tissue metabolomic data by genotype separated by age. The distances were calculated from pairwise comparisons from within each category (KO by KO and WT by WT) and across genotype (KO by WT). Comparisons are colored according to the legend. *P*-values from the MWU-test are shown for each organ. Boxplots colored in red show significantly higher beta-diversity at 3-weeks than 8-weeks with *P* < 0.001. C: PCoA plots generated on metabolomic (Bray-Curtis distance) and microbiome (UniFrac distance) data from fecal samples collected through the first 8-weeks of life. Plots are colored by genotype and week of sample collection separately. PERMANOVA F and *P*-values are shown for the genotype comparison overall. D: Metabolome and beta-diversity changes through the lifespan (3- to 8-weeks) by genotype comparison. Statistical significance was tested pairwise across genotypes at 3- and 8-weeks and is significant (*P* < 0.001) unless otherwise shown. MWU, Mann-Whitney U test; PCoA, Principal co-ordinate analysis; PERMANOVA, permutational multivariate analysis of variance.

Eight-week-old KO and WT mice (both male and female, n = 6–8 per genotype, per sex) and 3-week-old KO and WT mice (both male and female, n = 7–8 per genotype, per sex) were dissected and tissue samples were analyzed with LC-MS/MS metabolomics. Gallbladder, liver, duodenum, jejunum, ileum, cecum, colon, brain, kidney, and serum samples were collected from adult mice, but only cecum, gallbladder, ileum, liver, and serum were collected from weaned pups due to their small size. Principal co-ordinate analysis (PCoA) plots showed that tissue metabolome at 8-weeks was primarily separated by sample type, as expected, but also had significant differences between genotypes across the dataset as determined by PERMANOVA testing (F = 9.09; *P* = 0.001, Fig. 2A). Comparison of the Bray-Curtis distances between mice across genotype and within genotype enabled determination of the overall effect of the *Baat* KO on the tissue metabolomic data relative to variation within individuals of the same genotype (Fig. 2B). The gallbladder had the highest amount of metabolome variation at both ages (3- and 8-weeks). At 3-weeks, all tissue types (including serum) had significantly higher metabolome variation across genotypes than within, except for the ileum (Fig. 2B). At 8-weeks, a similar trend was found with the gallbladder, liver, duodenum, and ileum, having significantly different metabolomes across genotypes rather than within, but this difference was lost in the jejunum, cecum, and colon, in addition to the brain, kidney, and serum (Fig. 2B). Notably, metabolome variation across genotype within the same organ (KO-WT) was significantly higher at 3-weeks than 8-weeks, indicating that *Baat* gene KO had a greater overall effect on murine biochemistry after weaning than adulthood (Fig. 2B). Sex differences of the metabolome between KO versus WT were found to be minimal at 3-weeks but significant in the gallbladder, liver, and cecum at 8-weeks, though overall sex effects from the KO were minimal in the tissue metabolome data (supplemental Fig. S3).

Fecal samples were collected from KO, HET, and WT mice after weaning (3-weeks) to 8-weeks on a weekly basis and the microbiome and metabolome were analyzed. The metabolome data showed significant differences between the three genotypes as well as the expected change with age (Fig. 2C). The PCoA plots showed that separation of the *Baat*^*-/-*^ animals was particularly pronounced early in life. Analysis of the Bray-Curtis distances across genotypes through time verified this observation. At 3 weeks, the KO-WT and KO-HET comparisons showed the greatest metabolomic differentiation (MWU-test, *P* < 0.001). Over time this difference waned, and the metabolome of the KO animals became more similar to WT but remained significantly different than the within genotype variation at 8-weeks (Fig. 2B, *P* < 0.001). Sex effects in the fecal metabolome were again subtle, with significance only found across KO-WT comparisons at 6-weeks (supplemental Fig. S3).

We summed phospholipid abundances in tissue and fecal metabolomes to assess the difference between genotypes at both 3- and 8-weeks of age. Total phospholipids were higher in the fecal samples of KO animals in the first 3 weeks after weaning but then normalized into adulthood (supplemental Fig. S4). Three-week-old *Baat*^*-/-*^ mice had reduced phospholipids in the liver with a matching trend present in serum (*P* = 0.067), whereas the reverse trend was found in the cecum (supplemental Fig. S4). These findings indicate that KO animals may be excreting more lipids early in life due to lack of absorption into the bloodstream. In adulthood, however, this trend was not significant in any organ except for the gallbladder and serum (supplemental Fig. S4). Liver storage forms of vitamin A were detected (retinol and retinal) in the metabolome data from these animals. Retinal was significantly higher in the liver of WT mice than KO at 3-weeks; there was no significant difference in retinal or retinol between genotypes at 8-weeks (supplemental Fig. S5).

### Microbiome variation in KO, HET, and WT from weaning to 8-weeks

The microbiome data of the fecal samples (KO, HET, and WT) showed similar trends to the metabolome. Clearly present in the PCoA plot was a changing microbiome with age, and though not as strong as the metabolome, genotype was also significantly different overall (Fig. 2C, PERMANOVA *P* = 0.001). At 3-weeks, the KO-WT and HET-KO UniFrac distance comparisons showed the greatest microbiome variation, which was significantly different than within genotype variation, but this differentiation in microbiome beta-diversity was quickly lost and only subtle differences were seen at 8-weeks (Fig. 2D). Sex effects were also small in the microbiome data with significant differences in beta-diversity only found at 6- and 7-weeks (Fig. 2D).

**Fig. 3:**
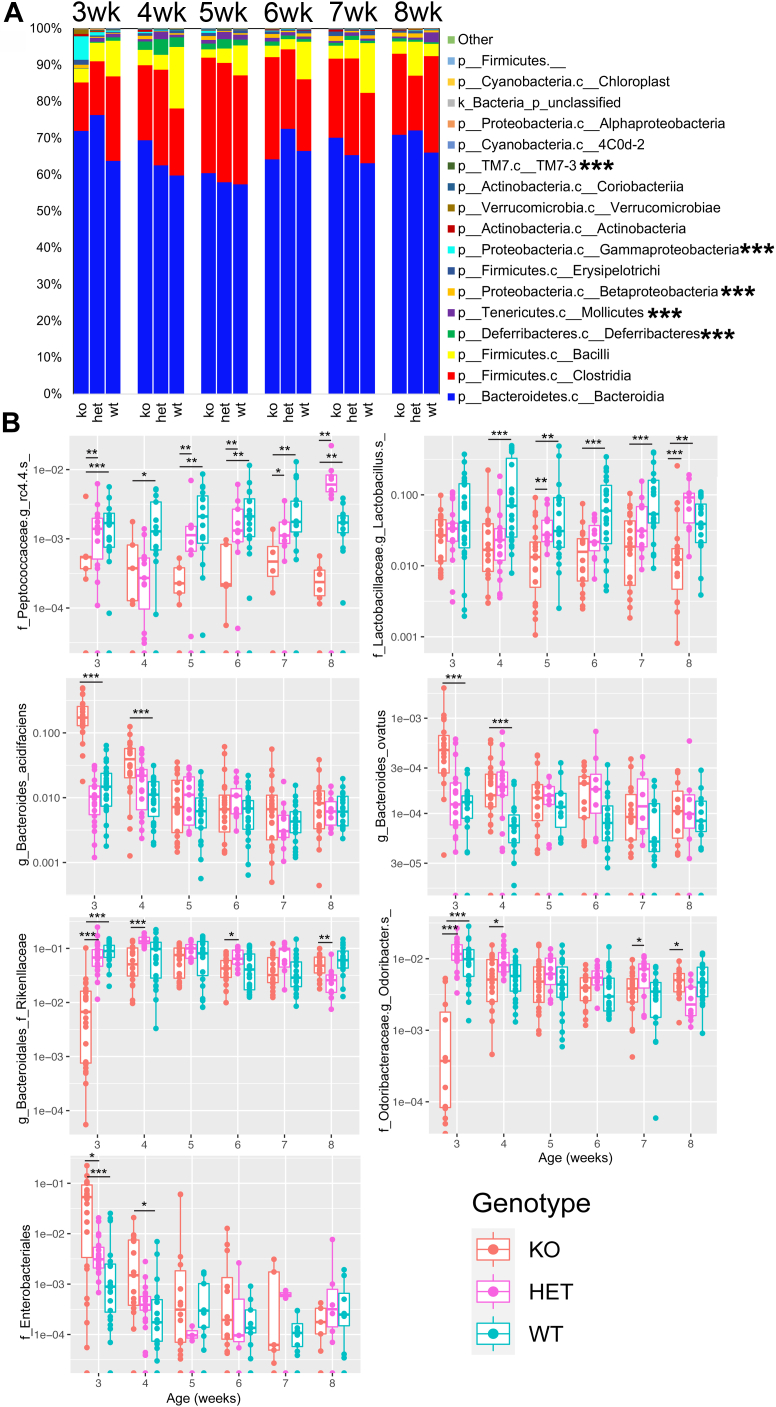
Fecal microbiome variation in *Baat* genotypes from 3- to 8-weeks. A: Microbiome bar plot at the Class level averaged from all mice within the KO, WT, and HET genotypes from 3- to 8-weeks. Significance testing at the family level is shown next to the bacterial class (∗ =*P* < 0.05, ∗∗ =*P* < 0.01, ∗∗∗ = *P* < 0.001, MWU-test). B: Boxplots of the relative abundances of different ASVs in each genotype (male and female combined) from 3- to 8-weeks. ASVs, amplified sequence variants; MWU, Mann-Whitney U test.

After the beta-diversity analysis showed that *Baat* gene deletion altered the murine gut microbiome, we used random forest (RF) machine learning to further determine genetic effects on the microbiome and prioritize which microbes were driving the differentiation. The overall RF classification error from the fecal samples collected from 3- to 8-weeks including KO, HET, and WT mice was 20.47% (supplemental Table S2). The KO genotype classification error was 14.7%, indicating that the microbiome reflected the genotype of the animals, especially those of the KOs, but not as strongly as expected. Class level analysis of the microbiome profiles through time showed little overall differences, except for several at the 3-week timepoint, most notably, Gammaproteobacteria (Fig. 3A). Ranking the amplified sequence variants (ASVs) most associated with mouse genotype identified several bacteria driving the microbiome variation (supplemental Table S2). A *Peptococcaceae* ASV, a *Lactobacillus* sp., *Bacteroides acidifaciens*, and *Bacteroides ovatus* were among the strongest classifiers (supplemental Table S2 and Fig. 3B). The two former organisms were elevated in the WT mice and remained so throughout their lifespan, while the latter two organisms were elevated in the KOs, exclusively in the first week after weaning. Conversely, a *Rikenellaceae* ASV and member of the *Odoribacter* genus were significantly decreased at three weeks but not after (Fig. 3B). This taxonomic analysis of the *Baat*^*-/-*^ genotype showed that the microbiome was altered in these animals, but not strongly, and the effects observed were most prominent in the first week after weaning.

### Variation of known BA in *Baat* KO mice

**Fig. 4:**
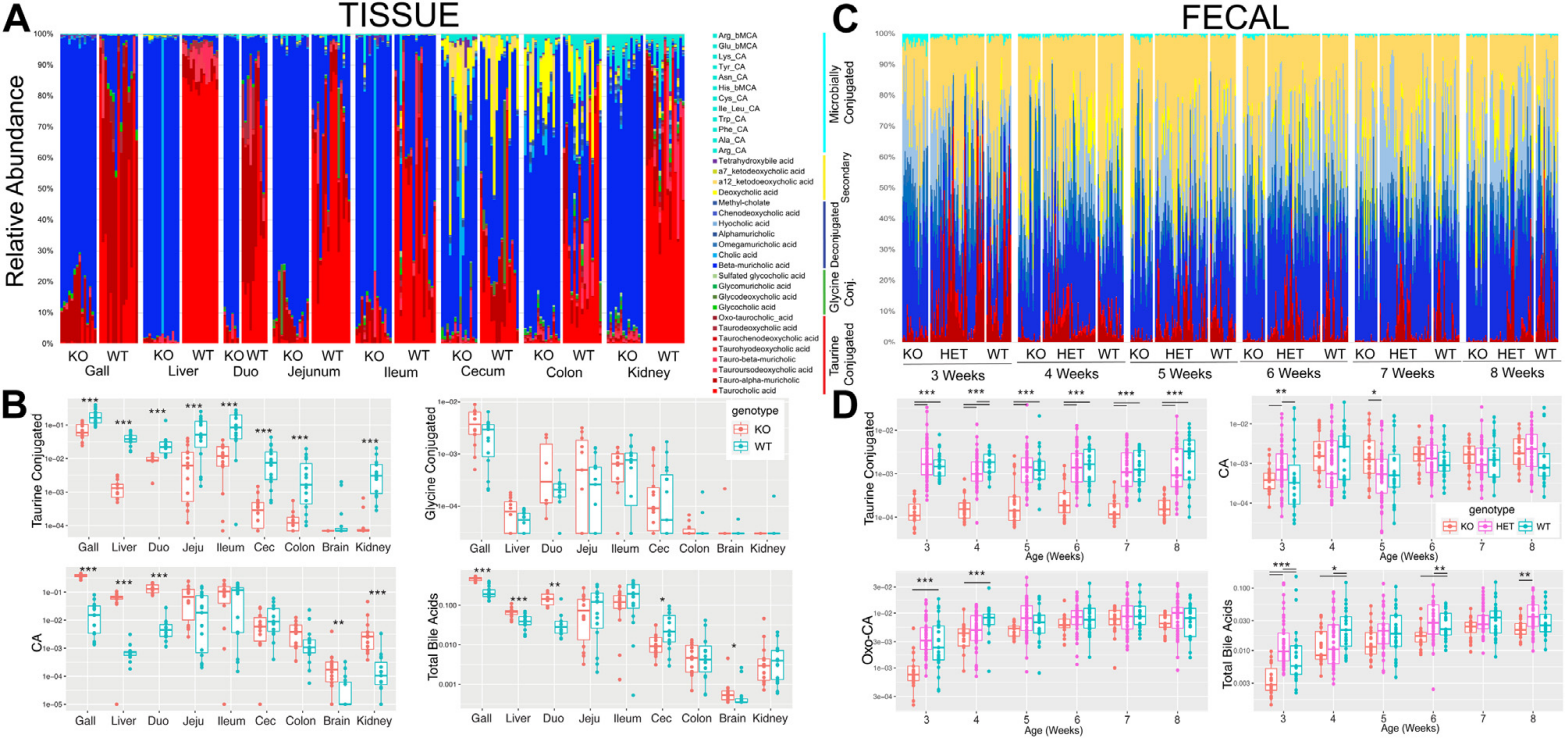
Known BA profiles in tissue and fecal samples. A: Relative abundance plots of known BAs present in the tissue samples of KO and WT animals. Each bar in the bar graph represents an individual animal separated by genotype and tissue. Bar plots are colored by the bile acid class with taurine conjugates shown in shades of red, glycine conjugates in green, deconjugated BAs in blue, secondary BAs in yellow, and MCBAs in teal. B: Boxplots of the normalized abundance of taurine/glycine conjugated BAs, CA, and total BAs by genotype. C: Relative abundance plots of known bile acids present in fecal samples per genotype, separated by age. D: Boxplots of the normalized abundance of taurine-conjugated BAs, CA, oxo-CA, and total BAs in the fecal samples by genotype. Significance is tested between KO-WT and/or KO-HET using the MWU-test. ∗∗∗ = *P* < 0.001, ∗∗*P* < 0.01, ∗*P* < 0.05, Duo = duodenum, Jeju = jejunum, Cec = cecum. BA, Bile acid; CA, cholic acid; MCBA, microbially conjugated bile acid; MWU, Mann-Whitney U test; oxo-CA, oxidized CA.

We explored the abundances of known BAs in the fecal and tissue samples of the different genotypes at 8-weeks. Collectively, taurine-conjugated BAs were significantly reduced in both the tissue and fecal samples of *Baat*^*-/-*^ (*P* < 0.001 for all tissues except brain, MWU-test *P* < 0.001; all fecal samples, Kruskall-Wallis and Dunn test post-hoc, Fig. 4A, B). The mean fold difference in the abundance of taurine conjugates varied by organ, being only 2.6x higher in WT gallbladder, 27x higher in liver, 8x higher in ileum, 28x higher in cecum, 30x higher in colon, and 36x higher in the kidney. By our retention time analysis with standards, tauro-omega-muricholic acid and TCA were the most abundant conjugates in our animals. However, the omega-, alpha-, and beta- forms of tauromuricholic acid (TmCA) could not be easily separated with our untargeted metabolomics workflow, thus, we collectively refer to them as TmCA henceforth (supplemental Fig. S6). TCA and TmCA were significantly more abundant in liver, ileum, cecum, colon, and kidney samples of WT animals (supplemental Fig. S7). Significance was not reached in the gallbladder or brain for both molecules and duodenum for TCA and jejunum for TmCA (supplemental Fig. S7). TCA and TmCA were significantly more abundant in all WT fecal samples at all timepoints (supplemental Fig. S8). Glycine-conjugated BAs, however, were not significantly different between KO and WT in any tissue and were not detected in the fecal samples (Figs. 4A–C, S7, and S8). CA dominated both the KO and WT animals compared to muricholic forms at 8-weeks and was significantly higher in the gallbladder, liver, and duodenum of KOs (supplemental Fig. S7). Statistical significance was lost in the lower regions of the GI tract but was also present in the brain and kidney. The muricholic form, though lower abundance overall, was significantly elevated throughout the GI tract of *Baat*^*-/-*^ animals (supplemental Fig. S7). Interestingly, CA did not show strong differences in the fecal samples, being slightly higher in KOs at 5-weeks (Fig. 4B). Lysocholic acid, a microbially conjugated bile acid (MCBA) detected in the fecal samples, was significantly higher in KO mice throughout the lifespan (supplemental Fig. S8). Other microbially modified BAs, particularly oxidized forms of cholate (Fig. 4D), were also highly abundant in the fecal samples and significantly reduced in KO animals at weeks 3 and 4 (Fig. 4D). Total BAs in 8-week-old *Baat*^*-/-*^ animals were significantly higher in the gallbladder, liver, and duodenum, but this difference was not seen in lower sections of the GI tract (Fig. 4B). Total BAs in the fecal samples showed the opposite trend, being more abundant in the WT animals, but only at 3-, 4-, and 6-weeks of age. This contrasting trend may indicate that even with more BAs in the gallbladder and liver in *Baat*^*-/-*^ mice, more are absorbed back into the bloodstream in the ileum than WT mice. Indeed, CA and CDCA were significantly higher in the serum of KO animals (supplemental Fig. S9).

Trends in the 3-week-old animals generally held in adults, albeit with some notable differences (supplemental Fig. S10). TCA was more abundant in WT than KO; however, some KO 3-week-old mice had significant amounts of TCA in their liver and ileum (supplemental Table S3 and supplemental Fig. S10). TmCA was more abundant in the 3-week-old animals and was again significantly higher in WT than KO. CA was more abundant in KO animals as found in adults. Quantification of TCA, GCA, and CA was performed in the liver and ileum of 3-week-old male and female mice. TCA was found to be 394.18 (+/-261.15) μmol/g tissue in the WT female ileum and 38.29 (+/-159.32) μmol/g tissue in KO ileum. CA was 74.82 (+/-1.50) μmol/g tissue in the WT ileum and 356.52 (+/-3.13) μmol/g tissue in the female ileum (supplemental Table S3 and supplemental Fig. S11). In addition, a tetrahydroxy-BA was more abundant in KO animals (tetrahydroxy-BA MS/MS supplemental Fig. S12), and there were also significantly more MCBAs in the younger animals, with heightened levels in KO gallbladder, ileum, and cecum (supplemental Fig. S10). MCBAs were not detected in serum of pups.

### Discovery of unusual BAs in *Baat*^*-/-*^ mice

A RF classification analysis was used to further explore the degree of metabolomic variation between the genotypes of adult mice and identify metabolites driving the variation. Due to the beta-diversity analysis showing that the greatest genotype difference in the tissue metabolome was found in the gallbladder and liver, RFs from these organs were used to identify the metabolites best separating the genotypes (Liver RF classification error = 0%). Of the top 30 metabolite features differentiating the liver metabolome that were known in the GNPS libraries, all six were BAs (supplemental Table S4). Other metabolites of interest identified from the tissue RFs from 8-week-old mice included elevated bilirubin in the livers of WT mice (MWU-test *P* = 0.001), elevated O-adipoylcarnitine in the liver and kidney of KOs (MWU-test *P* = 0.009, *P* = 0.0013, respectively), elevated 5-hydroxyindoleacetic acid in the colon of KOs (MWU-test *P* = 0.0048), as well as riboflavin and biliverdin elevated in the gallbladder of WT (MWU-test *P* < 0.0001; *P* = 0.00084, respectively).

**Fig. 5:**
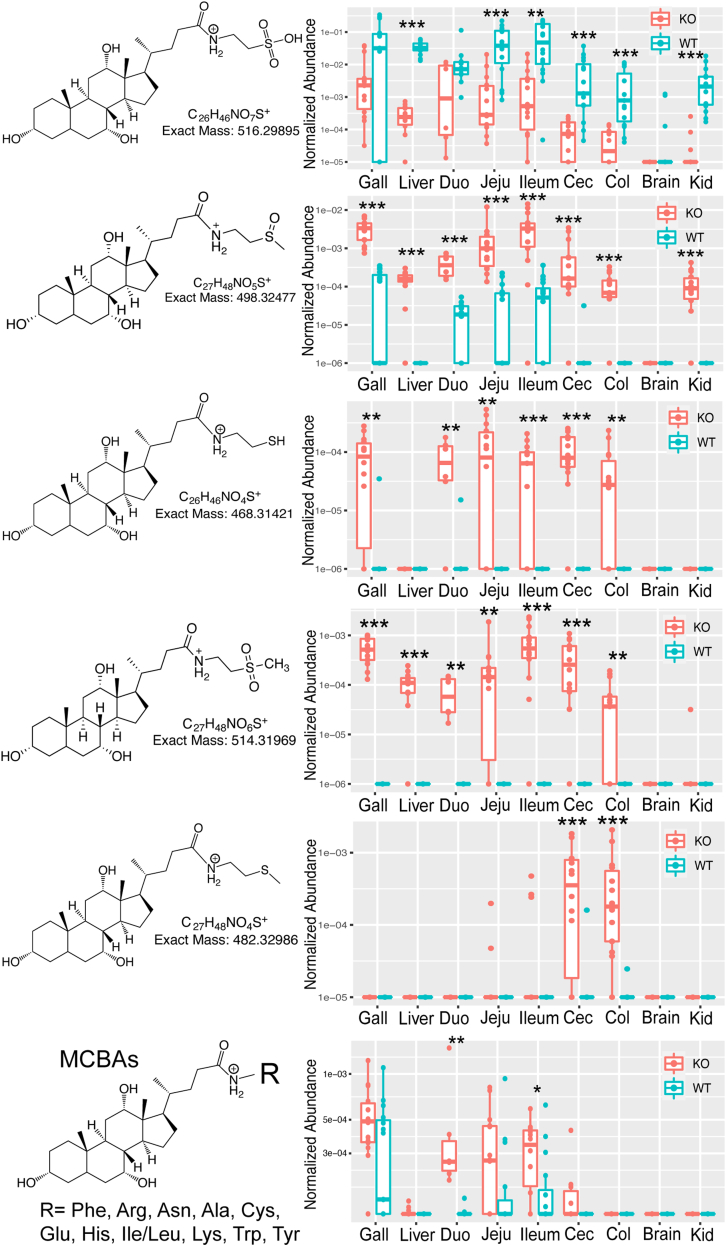
Unusual BAs detected in *Baat*^*-/-*^ mice. Boxplots of the abundance of each unusual BAs are shown in each tissue type by genotype according to the legend. Note that stereochemistry and position of oxidized BA on sterol core cannot be discerned with our methods. Significance was tested via MWU-test. ∗ = *P* < 0.05, ∗∗ = *P* <0.01, ∗∗∗ = *P* < 0.001, MCBA = microbially conjugated bile acids, Duo = duodenum, Jeju = jejunum, Cec = cecum, Col = colon, Kid = kidney. BA, bile acid; MWU, Mann-Whitney U test.

Inspection of the MS/MS data of genotype differentiating metabolites showed that many were also BAs but without annotations in the GNPS libraries. The MS/MS fragmentation patterns of many of these molecules had similar acyl-conjugated amino acid-like peaks analogous to that described in (7, 28), but these could not be initially identified as known amino acids. Instead, the fragmentation patterns and follow-up pseudo-MS^3^ experiments indicated that these compounds were BAs conjugated with cysteamine with oxidations and a methylation on the sulfur atom, producing a similar, but not identical, structure to taurine (supplementa Fig. S13 and S14). Some of the most abundant unique BAs included *m/z* 468.3130, which is conjugated with cysteamine, *m/z* 482.3299, which is conjugated with S-methylcysteamine, *m/z* 498.3247, which is conjugated with an oxidized S-methylcysteamine, and *m/z* 514.3197, which is conjugated with C_3_H_9_NO_2_S+H^+^ representing a double oxidation on the sulfur group of S-methylcysteamine. Interestingly, the S-methylcysteamine conjugate was only found in the lower sections of the GI tract and the fecal samples, possibly associating this molecule with the microbiome. The singly oxidized S-methylcysteamine conjugate (*m/z* 498.3247) was the most abundant overall, reaching similar levels as TCA in the KO mice and in some cases reaching TCA levels present in WT (colon). There were many other unusually conjugated BAs in the *Baat*^*-/-*^ animals, and some were putatively identified (Fig. 5). This included the cysteinocholic acid (Cys-CA), which was exclusively detected in KO animals at 8-weeks, particularly in the gallbladder (supplemental Fig. S15). This contrasts with other MCBAs that were found in both KO and WT mice at both ages. Cys-CA may represent another unique BA produced in KO mice because of a nonfunctional BAAT protein. Spectral searching of the various cysteamine conjugates against the GNPS public mass spectrometry data with MASST (29) showed that they were not unique to *Baat*^*-/-*^ animals and they were also found in other rodent and mammalian samples, but not humans (supplemental Table S5). Interestingly, Cys-CA and the *m/z* 468.3130 conjugate did not have any hits in the GNPS database, indicating it may be unique to *Baat*^*-/-*^. The unusual BAs made up over 25% of total bile in KO animals in the cecum and colon (supplemental Fig. S16). In the 3-week-old mice, these unusual conjugates were also detected and highly abundant in the KO animals; however, the cysteamine (*m/z* 468.3130) and Cys-CA molecules were not detected (supplemental Fig. S17). Collectively, these unusual BAs remain only putatively identified as standards are not available and isomers of the proposed conjugates are possible. Other backbone BAs besides CA were also identified with these unique conjugations.

Another set of BAs that were highly abundant in adult KO mice were unconjugated tetrahydroxy BAs. Interestingly, an oxidized CA derivative (oxidation position at 3α, 7α, or 12α unknown) was also highly abundant in the tissue samples but the genotype differences for this BA were not as strong as the tetrahydroxy forms. Finally, MCBAs (n = 11 unique amino acid conjugates) were also found in these animals including in the gallbladder and were significantly more abundant in the KO mice (Fig. 5).

### Exploring the source of BA conjugates in *Baat*^*-/-*^

**Fig. 6:**
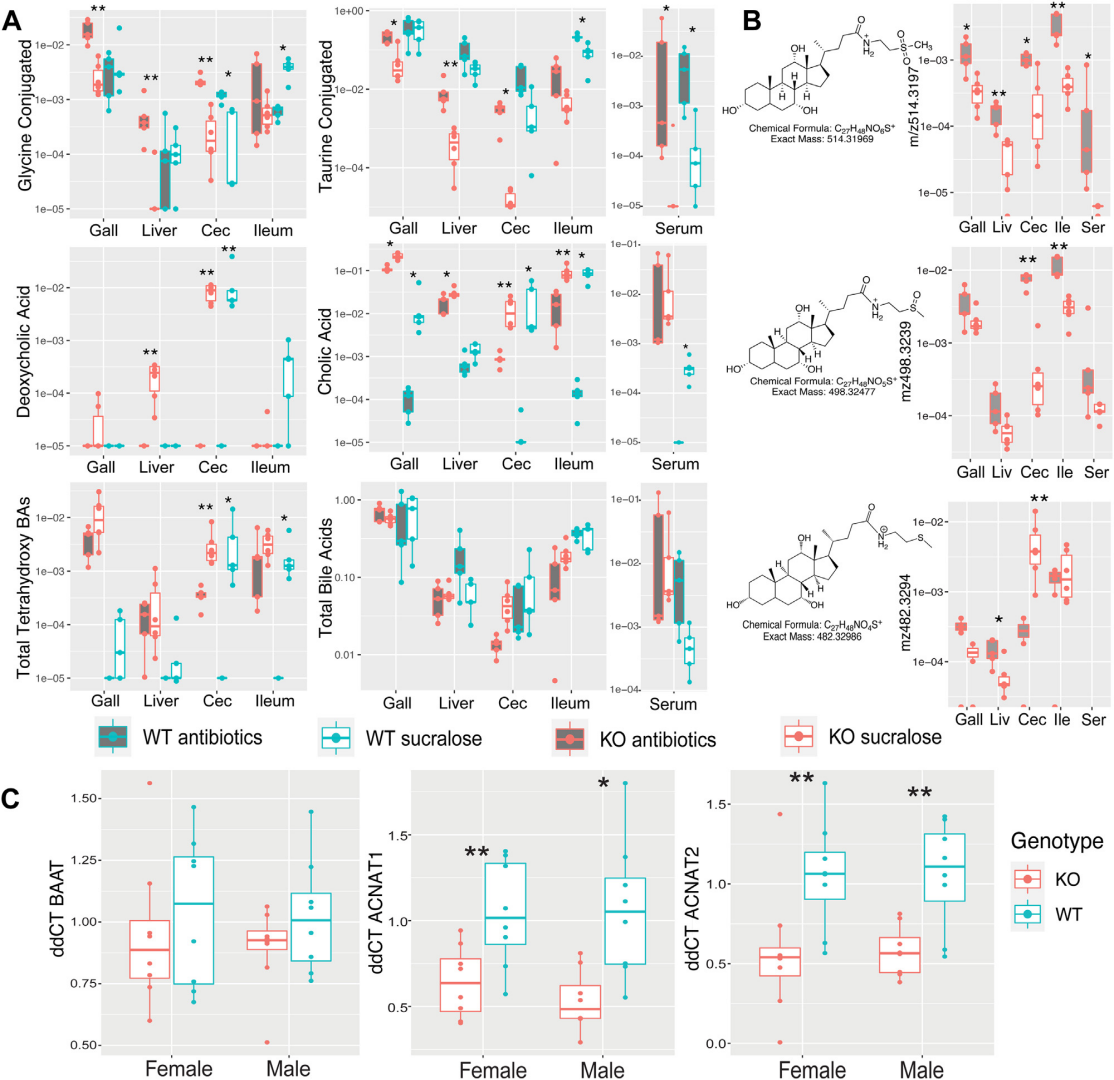
Effect of broad-spectrum antibiotic treatment on BA abundance in WT and KO mice. A: Boxplots of the normalized abundance of total glycine/taurine conjugated BAs, total BAs, and other BAs of interest in gallbladder (Gall), liver, cecum (Cec), ileum, and serum (if detected) samples. Results of significance with or without antibiotics is tested with the MWU-test (∗ = *P* < 0.05, ∗∗*P* < 0.01, n = 5–6 per group). B: Boxplots of unusually conjugated BAs in WT and KO mice. Boxplot show the normalized abundance of conjugated BAs of interest in *Baat*^-/-^ mice only as their abundance was negligible in WT animals. Cec = cecum, Ile = ileum, Ser = serum. C: Gene expression as ddCT from *Baat*, *Acnat1*, and *Acnat2* in 3-week-old male and female mouse livers. BA, bile acid; MWU, Mann-Whitney U test.

The presence of taurine- and glycine-conjugated BAs in *Baat^-/-^* mice led us to explore if they were of microbial origin. The gut microbiota are known to conjugate BAs with a wide variety of amino acids including glycine (6, 7). Thus, we first tested whether the MCBA producing bacterium *E. bolteae* BAA-613 could also synthesize TCA. Culture of *E. bolteae* in reinforced clostridium medium exposed to CA and taurine resulted in efficient production of TCA (supplemental Fig. S18), creating the potential that both GCA and TCA in *Baat*^-/-^ mice were produced by the microbiome. To further explore this, 8-week-old KO and WT mice (n = 5–6 per group, combined genders) were treated with a cocktail of five antibiotics for 14 days. Taurine-conjugated BAs were significantly increased in the GI tract of KO animals when given antibiotics (180x higher in cecum, Fig. 6A). Glycine-conjugated BAs, though far less abundant than taurine conjugates overall, were also significantly increased in the gallbladder, liver, and cecum of antibiotic-treated KO mice. Accordingly, the opposite trend was observed with CA, where antibiotics significantly decreased its abundance in gallbladder, liver, and cecum of KO animals, with a similar effect on CA in the WT (Fig. 6A). These results show that, as expected, the antibiotic treatment was reducing the bacterial deconjugation of TCA and GCA by bile salt hydrolase. Similarly, production of deoxycholic acid was completely depleted by antibiotics in both WT and KO (Fig. 6A). Thus, bacteria were not the likely source of the TCA and GCA in *Baat*^-/-^ mice, as microbial BA enzyme activity was highly depleted in this experiment, but these molecules remained in the KO mice and even increased in abundance.

The unusually conjugated BAs primarily present in KO animals were also analyzed for the influence of antibiotics on their abundances. Similar to TCA and GCA, antibiotics significantly increased the unusual BAs (*m/z* 514.3197 and *m/z* 498.3248, Fig. 6B). The S-methylcysteamine conjugate (*m/z* 482.3299), however, showed the opposite trend and decreased with antibiotic treatment. This supports its association with the microbiome described above, indicating that gut microbes may further modify these unusual BAs. Analysis of molecular networks of these unusual conjugates shows many other related molecules with the same, unique conjugation, albeit with different BA core structures also show the same trends in relative abundance as these representative BAs (supplemental Fig. S19).

After evidence from the antibiotic experiment indicated that the glycine, taurine, and the unusual BA conjugations detected in *Baat*^-/-^ mice were not of microbial origin, gene expression assays were used to assess if the known BA conjugation genes Baat and *Acnat1* and the gene with unknown function *Acnat2* were expressed in 3-week-old KO and WT animals. Primers for cDNA of *Baat* that targeted the N-terminal region ahead of the gene deletion in exon 2 (see supplemental methods and data) successfully produced an amplification product from both KO and WT, though the expression levels were not significantly different (Fig. 6C). *Acnat1* and *Acnat2* were also expressed as measured by cDNA amplification with their specific primers, and in both cases, their expression levels were significantly higher in WT mice (Fig. 6C). While cloning and characterization of the enzymatic properties of *Acnat* and *Acnat2* were not performed in this study, these results show that these genes were expressed in *Baat*^-/-^ mice.

## Discussion

BAAT-mediated amino acid conjugation with either glycine or taurine is the final step in BA synthesis. The enzyme is ubiquitous across the Mammalia and present in amniotes (18). By its very biochemical and evolutionary nature, BAAT is likely a fundamental enzyme for proper mammalian GI function. Though KOs of almost all genes in the BA synthesis pathway have been studied (30), the role of the BAAT enzyme in GI physiology has received little attention in KO mouse models. Similar to a recent study of this same KO mouse strain by Alrehaili *et al.* (31), C-terminal exon deletion has strong effects on BA chemistry, the gut microbiome, and host physiology. The untargeted metabolomics and microbiome data approach used here showed that effects of the gene deletion are most prevalent in the first weeks after weaning. KO mice had greatly reduced taurine-conjugated BAs as expected, creating a GI tract highly enriched in CA, but these primary BAs were still present, abundant in some individuals, and there was no effect seen on glycine conjugation. Furthermore, KO mice contained an abundance of unusually conjugated BAs that have structures that mimic taurine (18) and abundant MCBAs only recently described (28). Results from this study highlight our poor understanding of mammalian BA conjugation in general, due to the surprising presence of glycine and taurine conjugates and the diversity of unexpected acyl-conjugated BAs in *Baat*^-/-^ animals.

Similar to that reported in Alrehaili *et al.* (31), *Baat*^-/-^ mice were underweight after weaning indicating that BA conjugation is important for proper growth and physiology early in mammalian development. Here, mice of both sexes quickly exhibited catch-up growth, such that after 4-weeks in females and 5-weeks in males, they were not different from WT. In contrast, Alrehaili *et al.* (31) reported catch-up growth at 7-weeks. Histopathological analysis of livers from WT and KO mice at 3-weeks-old demonstrated a decrease in hepatocellular glycogen storage in KO mice. Furthermore, 3-week-old mice had smaller livers and subcutaneous fat pads. We therefore hypothesize that the effects observed in pups after weaning is related to feeding on breast milk as the primary energy and nutrient source. Because murine breast milk contains high amounts of fat, higher than mouse chow given to adults, this may enhance the effects of the *Baat*^-/-^ which greatly reduces the fat-solubilizing TCA. Pups more dependent on the fat-enriched breast milk may suffer more from the lack of fat absorption, changes in metabolism, and energy depletion, manifesting as decreased PASH-stained granules, interpreted as glycogen stores, in the liver of *Baat*^-/-^ mice and lowered fat storage in other tissues. Supporting this hypothesis was the finding that total phospholipids were decreased in the liver of KO pups and increased in their feces, indicating that the animals were excreting more fat in their stool with less absorbed for metabolism. Alrehaili *et al.* (31) demonstrated that adult *Baat*^-/-^ mice fed high fat diet were protected from obesity and gained less weight than WT animals fed high fat. Collectively, these studies verify the importance of taurine conjugation for fat absorption in the murine gut during the weaning period and adulthood. The reduced vitamin A stores at 3-weeks, but not at 8-weeks, supports the importance of BA conjugation in absorption of this lipid soluble vitamin early in life but further measurements of the collective fat-soluble vitamins are required. Thus, similar to that observed in rare cases of genetic dysfunctions of the BAAT enzyme in humans (10, 15), this study further implicates taurine-conjugated BAs as important for the absorption of phospholipids and vitamin A in mammals.

*Baat*^-/-^ animals had starkly different metabolomes, being especially unique in the gallbladder and liver, and progressively less so in the distal GI tract. The metabolomic differences between genotypes were primarily driven by the high abundance of CA in place of taurine-conjugated BAs and the presence of unusual BAs in KO animals. There were many unexpected findings in these animals, particularly the mere presence of TCA and lack of an effect on glycine-conjugated BAs. BAAT is known to conjugate both CDCA and CA with either glycine or taurine amino acids (4, 32), with the latter conjugate being more abundant in mice and the former more abundant in humans, though there are differing reports on these ratios in the literature (1, 33). One would expect that a deletion in *Baat* would greatly reduce or eliminate glycine conjugation of CA, but GCA and other glycine-conjugated BAs were not affected in the KO animals. Gut bacteria have recently been shown to conjugate glycine to BAs in culture experiments (7), and here, we show for the first time that a gut microbe can conjugate taurine as well. This creates the possibility that the microbiome was responsible for the presence of GCA and/or TCA in *Baat*^-/-^ mice; however, antibiotics increased the abundance of these molecules in KO animals. If the microbiome was the sole source of GCA and TCA, then the heavy antibiotic treatment performed here, which readily reduced known microbial BA transformations such as deoxycholic acid production, would be expected to reduce or eliminate these molecules. Instead, antibiotics increased TCA and GCA in KO animals indicating the conjugations were likely of host origin, though a possible role of the microbiome cannot be excluded by these experiments. It is therefore more likely that TCA and GCA in *Baat*^-/-^ mice are sourced from acyltransferases *Acnat1* and/or *Acnat2*, which are known duplications of the *Baat* gene in mammalian genomes, intact in mice, but inactivated in humans due to mutation (18). *Acnat1* encodes an acyltransferase that efficiently conjugates long-chain fatty acids to taurine, while the function of *Acnat2* remains unknown (24). Both genes were expressed in *Baat*^-/-^ livers, though at a lower level than WT, indicating there may be some disrupted regulation in KO mice. Similarly, these two acyltransferases might be responsible for production of the unusual BAs in KO animals, which contained the same amine conjugation at the CA acyl site as seen in TCA and GCA but with unique structures that were quite diverse. For example, the abundant conjugate *m/z* 514.3197 (C_3_H_9_NO_2_S + H^+^) contains a putative S-methylcysteamine twice oxidized on the sulfur atom, which structurally mimics taurine. Cloning and substrate profiling of *Acnat1* and *Acnat2* were outside of the scope of this study, but data here supports the notion that these two enzymes may be responsible for the various conjugations of BA in these KO animals and in WT mice. The unique conjugates may explain the survival and little apparent pathology of KO mice in adulthood, as the taurine mimics likely increase the solubility of CA, substituting for the reduced TCA in the KOs and functioning to absorb dietary fats and vitamins similarly. These unique molecules are not exclusive to *Baat*^-/-^ mice, as they are present in public mass spectrometry data from other murine samples and some other mammals (though not humans). Their strong association with rodents in the GNPS database and absence in human datasets further supports the role or *Acnat1* and *Acnat2* in their production, because the human *BAAT* duplication is a pseudogene (*BAATP1*) with inactivating mutations, but the murine tandem *Baat* duplications are both intact (18). Regardless of their source, this study supports the notion that the mechanism of BA conjugation has yet to be fully determined in mammals. Discrepancies have existed in the literature for decades about the biochemistry of BAAT enzymes and the ratio of taurine/glycine conjugations in different species (18, 32, 34, 35). Results reported here indicate there are more diverse mechanisms of BA conjugation in mammals than initially thought, and other enzymes, such as ACNAT1 and ACNAT2, may contribute to BA synthesis. Clearly, more research is needed on the mechanisms of BA conjugation in mammals, especially considering the vital role of these compounds in human health and microbiome homeostasis.

The microbiome was also altered by the *Baat* gene deletion, particularly after weaning, but the overall effects were small and almost indistinguishable in adulthood, which was somewhat in contrast to the findings of Alrehaili *et al.* (31) that show significant differences at 10 weeks of age. A microbial-associated metabolite in the colon 5-hydroxyindoleacetic acid was also altered due to the gene deletion. One would expect, considering the extensive literature on the antimicrobial properties of BAs and their role in shaping the gut microbiome (1, 36), that a large reduction in the amount of taurine-conjugated BAs and massive increase in the abundance of CA in the guts of KO animals would result in a highly altered gut microbiome. These buffered effects on the microbiome may also be due to the BA shift in KO animals to the unusual conjugates that might restore some function of TCA. One of the more notable effects was an increase in the abundance of *Bacteroides* spp. in early life. Future studies on the effects of the *Baat* KO on bile salt hydrolase activity and the growth of *Bacteroides* species warrant further investigation.

Collectively, this study shows that although the KO animals have reduced fecundity, *Baat* is not an essential gene for survival of mice, likely due to the finding that it is not the only BA amidating enzyme. Nevertheless, deletion of the C-terminal portion of BAAT results in major shifts in BA profiles, disrupts phospholipid metabolism, and creates a microbiome dysbiosis in early life. Though their source is unknown, the molecular mimics of taurine identified in these animals represent a sort of rescued biochemistry with the potential to overcome ablation of a gene believed to be highly important for proper GI function. The increased MCBAs detected in *Baat*^-/-^ animals, especially in the first weeks after weaning, may also provide some degree of molecular substitution for the reduced taurine-conjugated BAs. Further research on BA conjugation in mammals, performed by both the host and its microbiome, will lead to a better understanding of the role of these highly abundant biomolecules in the GI tract and how they enable chemical crosstalk between these two mutualistic biological entities.

## Data Availability

Microbiome sequencing data is publicly available on Qiita (qiita.ucsd.edu) under Qiita study ID #13781 and at the European Nucleotide Archive under accession ERP140746. Raw mass spectrometry data is publicly available at massive.ucsd.edu under ID MSV000088761. All network links on GNPS are listed in supplemental Table S6.

## Supplemental Data

This article contains supplemental data.

## Conflict of Interest

The authors declare that they have no conflicts of interest with the contents of this article.

## Supplemental data

Supplemental Figures

Supplementary Genetics
