# Supplementary material for: Baat Gene Knockout Alters Post-Natal Development, the Gut Microbiome, and Reveals Unusual Bile Acids in Mice
Source: J Lipid Res. 2022 Oct 13;63(12):100297. doi: 10.1016/j.jlr.2022.100297 (PMC9679037; doi:10.1016/j.jlr.2022.100297)
Supplement: Supplemental Figures [file mmc1.docx]

**Supplementary Figures**

***Baat* gene knockout alters early post-natal development, the gut microbiome, and reveals unusual bile acids in mice**

Kerri A. Neugebauer^1^, Maxwell Okros^1^, Douglas V. Guzior^1,2^, Jeremiah Feiner^1^, Nicholas J. Chargo^3^, Madison Rzepka^1^, Anthony Schillmiller^1^, Sandra O’Reilly^3^, A. Daniel Jones^1^, Victoria E. Watson^4^, James P. Luyendyk^4,5^, Laura R. McCabe^3^, and Robert A. Quinn^1^*

**Supplementary Figures**

**
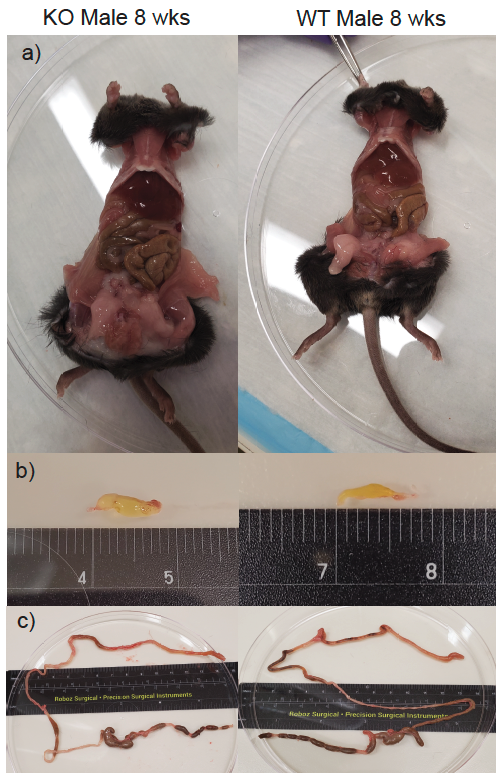
**

**Fig. S1.** Images of KO and WT male mice at necropsy. a) whole animal with GI tract revealed, b) gall bladder, and c) GI tract.

**Fig. S2.** Hematoxylin and eosin stains of liver and ileum sections from *Baat^-/-^* and WT female mice at 8-weeks-of-age. Scale bar is approx. 200μm.

**Fig. S3.** Beta-diversity variation between KO and WT based on gender in the tissue and fecal samples. For the metabolome (a-c) the Bray-curtis distance is calculated and for the microbiome (c-d) the UniFrac distance. The data is split into tissue 3-week and 8-weeks of age (a-b) and fecal samples (c-d) through the lifespan. Significance indicates that the multivariate data variation between KO and WT was higher for one gender compared to the other. P-values are calculated from the Mann-Whitney U-test.

**Fig. S4.** Total phospholipids in mice from this study by genotype. a) Phospholipid normalized abundance in fecal samples through the lifespan by genotype. b) Phospholipid normalized abundance in tissue samples mice at 3-weeks and c) 8-weeks. Significance was tested with the Mann-Whitney U-test *=p<0.05, **=p<0.01, ***=p<0.001

**Fig. S5.** Normalized abundance of vitamin A (retinal and retinol forms as detected) in the liver of mice at different ages by genotype. Significance was tested via Mann-Whitney U-test, with p-values shown.

**Fig. S6.** Tauromuricholic acid retention time standards comparison with mice gallbladders.

**Fig. S7.** Normalized abundance of known bile acids in WT and KO mice at 8 weeks of age. Significance was tested via Mann-Whitney U-test. *=p<0.05, **=p<0.01, ***=p<0.001

**Fig. S8.** Normalized abundances of known bile acids in fecal samples of mice throughout lifespan by genotype. Significance was tested via Mann-Whitney U-test. *=p<0.05, **=p<0.01, ***=p<0.00

**Fig. S9.** Known BA normalized abundances in serum of 8 week old mice by genotype. Significance was tested via Mann-Whitney U-test. *=p<0.05, **=p<0.01, ***=p<0.001

**Fig. S10.** Normalized abundance of known BAs in tissues of 3-week-old mice, separated by genotype.

**Fig. S11.** Boxplots of the concentration of TCA, GCA and CA per mass of tissue in the liver and ileum of male and female KO and WT mice.

**Fig. S12.** Retention time profile and MS/MS fragmentation of tetrahydroxy-BA detected in mice from this study.

**Fig. S13.** MS/MS fragmentation patterns and putative structures of unknown bile acids detected in *Baat^-/-^* mice.

**Fig. S14.** Pseudo-MS^3^ fragmentation patterns of unusual bile acids detected in *Baat^-/-^* mice. The acyl-conjugated fragment was isolated in the mass spectrometer after in source fragmentation and selected for MS/MS revealing peaks from the free conjugate. Putative structures, ppm errors, and chemical formulas for fragments are shown. Top: TCA, middle: *m/z*514.3197, bottom: *m/z*498.3248

**Fig. S15.** Comparison of cysteinocholic acid to all other MCBAs in tissue samples of 8-week-old mice. Significance was tested via Mann-Whitney U-test, with p-values shown.

**Fig. S16.** Percent of total BAs made up of the unusual conjugates in mouse tissue samples by genotype.

**Fig. S17.** Normalized abundance of unusual bile acids in the tissue of 3-week-old mice by genotype.

Media Only

**Fig. S18.** TCA production by *Enterocloster bolteae* BAA-613 after culture in RCM for 24 hours with or without CA and taurine.

**Fig. S19.** Three molecular networks of unknown BAs from 8-week-old murine tissue samples from the antibiotic treatment experiment. Molecular networks show conjugates related to those with structures shown (node outlined in blue) as determined by the cosine score. The network nodes are shown as pie charts based on the total abundance in the antibiotic treated or control animals. Outlines of the nodes denote presence in KO, WT, or both groups, and whether they are known BAs based on hits in the GNPS libraries, according to the legend.

**Supplemental Table Legends**

Table S1 – Variable importance plot from the fecal microbiome random forest classification based on genotype through time. Each amplified sequence variant is ranked based on its mean decrease in accuracy contribution to the RF classification. Also shown is the confusion matrix of the RF classification based on genotype for 8-week-old and 3-week-old mice with the out-of-bag error (OOB) and classification error within groups.

Table S2 – Quantification in µM/g tissue and +/- standard deviations of CA, GCA and TCA in livers and ileum from mice from this study.

Table S3 – Variable importance rank and confusion matrices from liver metabolomic data of 8-week-old mice. Details are shown as described in Table S1. Metabolome features are ranked by their mean decrease in accuracy. OOB= out-of-bag error of the RF classification.

Table S4 – GNPS output after MASST searching each unique bile acid conjugate from the KO mice. Tables a-d are each individual metabolite according to their parent mass. The columns show details of the search result including the dataset for which the spectrum was found, cosine score and peak matching of the hit. Also provided are links to the files, spectra and MASSive datasets on GNPS for each MASST hit in each public dataset.

Table S5 – Mouse sample size numbers for each treatment and gender in the various experiments from this study.
