# Supplementary material for: Baat Gene Knockout Alters Post-Natal Development, the Gut Microbiome, and Reveals Unusual Bile Acids in Mice
Source: J Lipid Res. 2022 Oct 13;63(12):100297. doi: 10.1016/j.jlr.2022.100297 (PMC9679037; doi:10.1016/j.jlr.2022.100297)
Supplement: Supplementary Genetics [file mmc2.docx]

**Supplementary Data – Genetic Information**

***Baat* gene knockout alters early post-natal development, the gut microbiome, and reveals unusual bile acids in mice**

Kerri A. Neugebauer^1^, Maxwell Okros^1^, Douglas V. Guzior^1,2^, Jeremiah Feiner^1^, Nicholas J. Chargo^3^, Madison Rzepka^1^, Anthony Schillmiller^1^, Sandra O’Reilly^3^, A. Daniel Jones^1^, Victoria E. Watson^4^, James P. Luyendyk^4,5^, Laura R. McCabe^3^, and Robert A. Quinn^1^*

**Below is the predicted amino acid sequence from the WT and KO *BAAT* gene.**

**Wildtype:**:

MAKLTAVPLSALVDEPVHIQVTGLAPFQVVCLQASLKDEKGNLFSSQAFYRASEVGEVDLEHDPSLGGDYMGVHPMGLFWSLKPEKLLGRLIKRDVMNSPYQIHIKACHPYFPLQDIVVSPPLDSLTLERWYVAPGVKRIQVKESRIRGALFLPPGEGPFPGVIDLFGGAGGLMEFRASLLASRGFATLALAYWNYDDLPSRLEKVDLEYFEEGVEFLLRHPKVLGPGVGILSVCIGAEIGLSMAINLKQIRATVLINGPNFVSQSPHVYHGQVYPPVPSNEEFVVTNALGLVEFYRTFQETADKDSKYCFPIEKAHGHFLFVVGEDDKNLNSKVHANQAIAQLMKNGKKNWTLLSYPGAGHLIEPPYTPLCQASRMPILIPSLSWGGEVIPHAAAQEHSWKEIQKFLKQHLLPDLSSQL-

**Mutant mouse strain C57BL/6NCrl-*Baat^em1(IMPC)Mbp^*/Mmucd (*Baat^-/-^*)**:

MAKLTAVPLSALVDEPVHIQVTGLAPFQVVCLQASLKDEKGNLFSSQAFYRASEVGEVDLEHDPSLGGDYMGVHPMGLFWSLKPEKLLGRLIKRDVMNSPYQIHIKACHPYFPLQDIVVSPPLDSLTLERWYVAPGVKRIQVKESRIRGALFLPPGPRPRCWHPFCMHWSRDWTFYGY-

Predicted protein sequences of wildtype and mutant *Baat* after CRISPR deletion of exon 2. The 22 amino acids of nonsense coding after the deletion and a premature stop are shown at the C-terminus of the knockout highlighted in pink. Highlighted in Red is the forward genotyping primer.

Amino acid sequence of full length *Baat* gene with exon and introns. Bold and underlined is the CRISPR gene deletion in the knockout animals. Highlighted in teal are the RT-qPCR primers for the *Baat* gene used in this study upstream of the CRISPR deletion. Highlighted in red is the forward genotyping primer and in green is the reverse primer to identify the knockout and wildtype.

>4 dna:chromosome chromosome:GRCm39:4:49489422:49506557:-1

GTCTCTTCTCTGGGTTCAGCCTACTTATATCTGGTTGAGGAGGGAAATACTAAGATATTT

TCCTCAGCTCTGACCGAATAGTCTGCATTTTTAAAAACTCTTCCATTATCTACAGTGTTG

TCAGAGCCTTGGTTTGAGAGTCTCTGAGAAGTCCTGGGCATCTGTGCTGACCGACAGGGC

CTCCTTCTCTAGAGCACACCACGTTCCTGAGGGTTGCTGTAAAACTACTGTAAGTGATTA

CTACCTCTGCCTTTCCTATGCAGCTTATCCTTTAATGAATTGCTCTGACGCCTGGTAGCT

CTCTTATCTATTATCTCTTAGCTCAGCGAACTCTTGATAAGTACATGAAGTTATCATTAT

CTACCCGCAGAAAATCTTACATCACTTGAAAAACAGTGATATAAGAATCCTATGTTTACC

AGATTTTCCTCTATTTGTCATATTTCATAGATAAACCTCATAAAACTTTTATTTTGAACA

GGCTATATAGAGTTTTAACTTTTCCTAAGTTCACATATGTATCTATGTAAGTGATTAAAT

TTTGAAAAGGGCCTTTATATTTACCCAGATAATAAATGTGTTATTGCTCTTCATTTCTTG

AAGTAGTATTTACTGAAAATGTATTTTCCCCATTTCCATTCCCCCATGGGACTTTCTTTC

TTTCTTTCTTTCTTTCTTTCTTTCTTTCTTTCTTTCTTTCTTTCTTTCTTTCTTTCTCTC

TCTCTCTCTCTCTCTCTCTCTCTCTCTCTCTCTCTCTCTCTCTCTTTCTTTCTTTCTTTC

TTTGGTTTTTCGAGACCAGGCTGCCTCTGCCTCCCAAGTGCTGGGATTAAAGGCGTGCGC

CACCGCGCCTGGCTGGGATTTACTTTCTATAGCCTTCAGGTTTGATGTGTATATTTCATA

GGTAAGGAATTGTGAGCATTTTGTTTTATTTACTTGTTTTTCTCTTTCTTTTGAGCACTG

TGATTGAGGGGTTATTGTCTCCTGCTTTGTTTAATGTCTTTTTGGTAGGGCCAACTGTCA

TTTTATGTTTGTTCTTTTCCTCTGCAATGTTTACTTTTCTCTGGCTACATTAAAATTTGC

TCTCTGTGTTAAGAGTTTGATTATAATAAGAGCTGGGATTTCTGCTGGGTTTGTCCCCAT

ATTTGTTATACTTCTTGTGACTGTAGCATGGTGGTTTTCAGTGAATTGAGGGGAAATTCA

GCCCTAGTATTTTTTTTAAGATTTATTTATTATTATAAATAAGTACACTGTAGCTGTCTT

CAGTCATACCAGAAGTGGGCATCAGATCACATTACAGATGGTTGTGAGCCACCATGTGGT

TGCTGGGATTCAAACTCAGGACCTTTAGAAGAGCAGTCAGTGCTCTTAACTGCTGAGCCA

CCTCTCCAGCCCCTCAGGCCCTAGTTTTTAATAAGTATTTCCCCTTTGCTTATCTCTCAT

ATGTCTTTCTAAGGGGCCAATTATTAGAGAATATATTCAGTATATTAAGCCATAAATATT

TTCCTGTGTTTCCTGTGTCTCAAGTTCTCTAATCTTTTATCTTGATTTTTCCTCTTCATA

GTTTAGCTTGAGCTGTTCTTATGTCTGCTTGGTGCCTTTTATTTGCTGTCTGGTAATTCC

AACGTCTGGGCTATTTCTCTTTCTATAATAATGATTCTTTTTTTCTTGGATTCTATAATT

TTAAATAACTTTTATGCAACTCAATGAAAAATAAATCCTGCGTGTGTTGTATATAAACAA

TATCTGAAAGGGGTAGGGCAATGATATTGCTTAGTGGGTAGAAGCACTTGCTGCCTACCT

AGCCTAACAAGCTGAGTTCCATGTCTGGGGGCCACAGGATTGAAGGAGAGAGTCAACTCC

CATCAACTATCACAGAAACTTTCTCTCTCTCTCTCTCTCTCTCTCTCTCTCTCTCTCTCT

CTCTCTCTCTCTCTCTCTCACACACACACACACACACACACACATGTACACGTATACACA

AATGCACACAGACCCACATATACACACATCCATACACTCACACACACATATCCATACACA

TACATACCCCCCCTCACACACACACAATTAATAAACAATTAAAAGAGCTTTGAACATGCT

TTGTTGAGACTTGGGGCTAATCAGTGTGACCTGAGCCAAGTGTGAGTTGGGTCTGGGACT

GTGACAGACTTATTAAGGTCAGTGCATCACTAACATCACTAATTTTATTAGTGGGGAGAG

TAAGTCACCTTTCTCATCAAGAGATGGGCTTCACAGCACTGGTGAGCCCCTTGTAGGTTC

TTTGGCTTTAACATCCATTTACTAGCCTTTTAAACTGAGGATTTCTGACGAGAGTTCTGG

ATCAGGGGTCACTAGGAAATTTGCTTGGCTCAGTGATAATACTCCTGGTTCGCTCAGCAT

AGGAACTTCTTTCTGCTCTTCCATCTTCATCCTCTGGAGGATGCTCTAAGTGAAGGCTCG

AAAGCCTCGGTGGCTTCATCTCAGATTGTGATCTGTGTCTTGGTCTTCTGGTGATGTCCT

GTAGACATGAACAGGTGGAGACAAGTTCTTAGTTTCTCAGAGTTCCATCAACCCAGACAG

CACCTATCTAAAGTACACCTCTTTTTCTGTTTTGTTTCTCATCTCTCTATGTGACTTCCT

TTCTGCTCCTTTATCATAAGTGAATATCAGAGAAGAAGGGACATTGTCCTGCTTCCTAGA

AGTAGGTCTTTTTCACCCCCTGGAACTTGATCCATTTAGATCTCTGGCATACTCAGCAAA

GTAAGTTAAGATGAAACTAACACAACAGATAAATAGATACAAATCTGATGCTGCAGTTTT

GGTTGTTAGGAGCAATGGTCACTCTTGTGTCAACATTTAAATGAAGACGATCAATAAATA

TTTCAGTAAGACTTCTGTTATGTTTTGAAGGTCTTAGCTGCTTCTAATTGTGTTTTATAA

CTGTATAAGTTCTAAGCCAAAGCAATGGTTATATTTCTTTTTTGTAAGGTGAAACATACA

GGTGCCTGTGCAAGACAGTAGTTAGCGGTGCCACTCATTTTGAAAAGGGAAATATTCAAT

CAATCATCCTGTTTTCAGCTTCACATCTCAATCCTGCTTTTGCCACAGTTGTGTGTGACG

CCAGATTGAGATCTCCTTACTGTCCTGACATCATCCATGCCGAGTTGGAGTGCTGTTTAA

ATATCTCACTTTTGTTAGTGTGCCCTCTGGCTTCTAGAAATCTGTTGAAATCTCTGAGGG

GTCTTGCATTACCAAATGATCACAGAATACTTGCAACAAATCAATGGAGAGTTAAGACTG

AATCCTGAGAGGTCTGACTCTAGTCCTTCCATCATTCATTAACCATATTACAACCTGATT

TCATTTCTGTTTCTTTCTGTTTTGAGGTTTTGGTGAAAATATTCCTGAAGAATTGTCCAA

GATTCCCTCTGCAAAAAATGGCCAAGCTGACAGCTGTTCCTCTCAGTGCTCTTGTTGATG

AGCCTGTGCACATCCAGGTCACAGGCCTGGCCCCCTTTCAGGTGGTGTGCCTTCAGGCAT

CACTGAAAGATGAGAAGGGAAACCTGTTTAGTTCTCAGGCCTTCTACAGGGCCAGTGAAG

TGGGTGAGGTAGATCTGGAGCATGACCCCTCACTTGGAGGAGACTATATGGGGGTCCACC

CCATGGGCCTTTTCTGGTCCTTGAAACCTGAAAAGCTATTGGGTAGATTGATAAAAAGAG

ATGTGATGAATAGCCCCTACCAAATCCACATAAAAGCTTGCCATCCATACTTTCCATTAC

AAGACATAGTCGTCAGTCCTCCCTTGGATAGCCTGACTCTGGAAAGGTGGTATGTGGCAC

CTGGGGTCAAGAGGATCCAGGTAAAGGAAAGCCGCATCCGGGGAGCCCTGTTTCTGCCTC

CAGGTGAGTAGAATCCCTCTGATTAGTGCCACCTCTGTTTCCTCTGTATGGGATGCCTTG

TACCTCTAGAAATTCAGCTTGGACATGAGTAATACAGAGTTCTCACCATCCTGCTGAGAA

GCTGGGTTAATTTTGTCTCAGAATAAGAAGTAGGTGATATTCTACTCTAATTCAAGTTTC

ACCCCCACATTCTGAACCTTGTCTGAGGCTAGGACTGGTTAGAGCTTGTGGGGTCCCTAT

ATTGTGAGGTTTCTGAAAATCTGGATGTCAGTTAAGTGGCTGGCTAAATGAAGACTCCAA

TAAAGCTATTGTCAGAAGATGTATCATGTTGACATTCATTTTAAGCTTGTTTAGATCATT

TAAATATGTTTATACTCAAAATATAGAACACTCTATTGTGCTTCAGGTCCAGGAGTTAGC

ACTGTTTTTTGTTTTGTTTTGGTTTATTTTTGTTTTTGATTTTTTTTTTTGTTTTTTTGT

TTTTTGTTTTTTTGCTCTTGTTTTCTGTTGTTGTTGCTTAGTATGACATCCCTGTTAAAA

ACATATACATTAGTGCAGGCCTTCTGACTCTGGCTGATATGCTAGCACCAGGTGTGTTAT

TTGCTATCTCCTGGGACATTGGATATGTAGAATCTCTGGCATCAAATGATCTTCTTGGGC

CCATAGGAGGGATGAGTTATATTTGGTCACAAAAGTTAAATTAGACATCAAACATTTCAG

GTCCTTTACTGTTCCCTTCTTTTCCACCCATCAGAAGATGCAAACTCATCTTTATTACTG

TACTTAATAGTAGTTTCCAATGTGAGATACATTGTTGTGGGTCTCCCTTTCCTTTGGGAC

CCCCTGCTTAGCAACTTTACTTAGGGTGATTGAAGGCTTATAAAGTTTTCTGGAACTTAG

GGCAGGGAAAACCAAGATGGGCAAAAGTCATTGGTTGGGGGCTTAGTGCACATGAAATGC

CTCATTAGCATGGGGAAGTGATTCAGGAATCTCAGGCTCTGGCTGGACAGGTTTTGTCAG

GAGAAAGGGAATTGATCATATAAACTAATTAGGGTTACCTGACTTCTCCGGATAAGGTCA

GAGAAGGAGAAAACCTGCTAATAAGGGAGTCTCCCATTTTGCTCCAAGCTCAGAGGGTAT

ACAATCATTGTGGACTTTGACCCTTACTAGCAGTGTTTTTACACATATTATGCCATCCCA

TTTCTTTTCTCAAGCTAAAGGACACATAGCCTGTTAAAAACTCCTCAGTGTCTCTTCATG

TGAAGCCCATCTTTAAATGGACATAGTTCATCTTCCTTCCCTAGAATCAAGTTTTCAAGA

AAGAAACATATAATTGTAGTGGGTGGGCTCTGGTTTAATTTAGAAATATCTGCCTGCACA

GAGTGAGTATGGACTGATGCTTGGACAGTCCTCTCTTTCACTGGGCTCTTACAGCAGGGA

GAGAGAGAGAGAGAGAGAGAGAGAGAGAGAGAGAGACAGAGAGAGAGAGAGAGAGAGAGC

TCTTCATTCATATCTGTTCACATGGAGATTTTATCAGTTGCTTTCTGAGTCGTTACCACA

GTATAACAGAGAAGGATATTTGGCCTTCCCCCCACAACCCCTGGTTCCTGCCATTAGCCT

TGAGACATAGGCACTTAAGGCAAGTGCTCCAAGACTGAGCTTCTGAGCTGTATCTCTTAC

AAGATATTTTAGATAGTGTAATCATTAAGATATCTTTGAGCCATGAACTTCAATTATGTA

GAGAGAACCTTTGTCTAACTAGGCCCTGTGAGGATGTGCTCTCAGGGACATGAAGATCTC

CAGCTAGGCTCCCTTCTTAAAGGTTCCATGTCCCCTACCTGCCCTTTGAAGGTAGCCTTT

GAAAGTTGGCCTTTGAAGGTATCAATCTATAGCACTTCTCATAGAAAAGCACATGTAAAC

AGAGATAGCATATTTAGTGTCAAATGTTGTGACCTTCTAGATTTCCTAAATCTCATTTGT

GTACCTTTTATTGATTAAGGAGTCCTGGCACAATTTGTATGGTATTTCTTACTCAAAGCC

TGTGGAAATTAATCCTAATCTGGGTCTCAAAGAGTAAATATGTCACTAAATGTGGCTTCT

AATAAAATCCATAGAAGTTATGTTCTACTACGTATTGACCAGTCTTCACTTAGCTTTAGA

CATGAGCTAACTATTTGATGCTCCATTTGAGAATTTGTGCTTTAATTGCAAGATTCAGTT

CTTCTTCTTGGCTTCCTATTCTCATTGGTGTTACCTGTAACTTTTTGATACCCTGCCTAT

GCTCTGGGAATGGGCCATGCTACCAGCCCCCTCCCAGCATTCCTTGAATCCATGGATAAA

AGACACACACACACACACACACACACACACACACACACACACAGTTGTTCATTTTCAACT

TGACATCTTGGCATAAAAGCTGGGTGCTAATGGAAAGCATGCTCTTATCATTACTCTTAG

CTCCACACCTCCCACCGGCCCTAACTTTAGTTTGTCAATAATCTTGTCCCTGCTAAACAT

CTCTGACCAACCTTGTATAGGAGCTCACCTATCCTCCTGAGACCTCACATGGTTCCTGGT

**TCTTCTCTCTCCAAAGCATGGTGACCTCCCTTCCCTTCCCTTGCCTTGCATCTGTGTACT**

**TTCTTCTCCAGGGACACGGAAGTCCTATCTGTACCTTCTACCCAGCAATTAGCCCATGGC**

**TTTCTTTTCTGAGAGATCAAGAGCCAATTGGGGAACAGGGCCTTAGCATCAGAACCACCC**

**CTATAGTTGGCTTCCTGCTTCTTTTTTTATGTGTTTTGTTTTGTGTTGCTTTTTTCCAGG**

**AGAAGGTCCTTTTCCAGGGGTCATTGACTTGTTTGGAGGTGCTGGTGGATTGATGGAGTT**

**CCGGGCCAGTCTTCTGGCAAGTCGTGGCTTTGCCACCTTAGCTCTGGCTTACTGGAACTA**

**TGATGACCTGCCTTCTCGACTGGAGAAGGTAGATCTAGAATATTTTGAAGAAGGTGTAGA**

**GTTTCTCCTGAGACATCCTAAGGTAATTTTAGGTTCATTAGTATGTGTATTAGTCAGCGT**

**TCTCTAGAGTCACAGAACTTATGGATAGTCTCTATATAGTAAATGAATTTATTAATGACT**

**TACAGTTTGCAGCCCAATTCCCAACAATGGTTCAGTAGTAGCTGTGAATGGAAGTCCAAA**

**GATTTAGTAGTTACTCAGTCTCACACATCAAGCAGGCGCAGGAGCAAGAGCAAGACTCCC**

**TTCTTCCAATGTCCTTATATTGTCTCCAGCAGAAGGTGTAGCCCAGATTAAAGGTGTGTT**

**CCACCACACCTTTAATCTGGGATGACCTTGAACTCAGAGATTTAGTCTTCTGGAATCCAT**

**AGCCACTCTGCCTCAAGATCTCCATACCAAGTTCCAGATCAGAAACTTCTTTCTTCAAGC**

**CTCCAGATAAGAGTCACTGGTGAGCCTTCCAATTCTGTATTGTAGTCCATTCCAAATATA**

**GTCAAGTTGACAACCAGGAATAGCCACTATTATCCACCCCTTGTCAACTTGACACAAATA**

**ATATCTCATGTTCACATGAAACAATAACAAGGTTGTGAATACGCCTAACATGATATAACT**

**ATCCCTCATACAATCGCAAACACATTTGTGAATTTACAATGGGGCAATGTCCCTTGGGAA**

**CATCCTTTTAGTGTCTCAACTTAAATACAAATTGATGTTAAAGAAATTGGAAGAAAGACA**

**AATATATTCGTTAGCAAAATAAGACAGGGACATTCATATTACTTTACAATCCTTGTTTCT**

**GCAACGGGTTATGTGGCCTTAATTGGTATTTATAACTACCTTCCTCTACCACCCATTCTG**

**TATTTCCTTCACCTTCAGCCAGCACCTCAGCAGGTCTTGGCTCTTTTCCTGGAGGATGGA**

**CCCATACCATCATTCCTGATGGGTCTGTGTCCTTTGGATTAGGC**TGTTGTAGTTTCCCAT

TGACTTTAATCACAGGACATGGTAGTACTAAGAGACGCCCTAAGGGATCTCCTACATTCC

AGACATAATCTTGCTTACCTCCATTGTGAAAAGGTAATCCAATTTCCCCATGGTAATCTG

GATCTATCACCCCTCCTAACACTGTTATTTCTTTTTTAGCCTGTTGGTTTAAGGGCATTA

GAAGCCCAAATGACCAGGGGGAAGTCTGAGCTTGCAGTTCATTGGGATATTTGTTGTAGC

TCCTGGTAGGAGCACTCCCTCCTCTGGAGCCAAAACCTCTAGGCCTGCAGAGCCTAGAGT

TATGGGTACAAGAAGCAAAAGTTTTCCTAGAGGGTCACTAGGAGTGATAGTGAGTGGAAC

TTTTCCACCCCTTGATTCCTGGACCCATGGATCCTGGTTATGGGGGAAACTGTACCATAT

ATCGAATACTGATTCGAGGCATATACTGCCTTCTGAAGAACTCTGCCCCAGCCTTCCATG

CTATTACCACCTAATTGGTGCTGCAACTGCATCTTCAAAAGGCTATTCCATCTTTCTAGC

AGACCAGCTGCTTCAGGTTGATGGGGAACATAGTAAGACCAGTGAATTCCATGATCGTGG

GCCCACTGTCATACTTCTCTGGCTGTGAAATGAGTTCCTTGGTCAGAAGCAATACTGTGT

GGAATACCATGACAATAGATAAGGCATTCTGTCAGTCTGTGGATGGTGGTTTTAGCAGAG

GCATTATGTGTAGGAAAGGCAAATCCATAACCAGTATAAGTATCTACTCCAGTAAGAACA

AAACGCTGTCCTTTCCACAAAGGAAGTGGTGCAATATAGTCAACCTGCCACCAGGTTGCT

GGCTGGTCACCTCGAGGAATGGTGCCATATCTGAGTCTCAGTGTTGGTTTCTGCTGTTGG

CAGATCTGGCATTCAGCAGCAACTGTAGCCAGGTCAGCCTTGGTGAGTGGAAGTCCATGT

TGCTGAGCCCAAGCATAACCTCCATCTCGACCATCATGGCCACTTTGTTCATGTGCCCAT

TGAGCAATGACAGGGATGGCTGGGGAGAGAGGCTGACTGTCCACAGAACGGGTCATCTTA

TCCACTTGATTATTGAACTCCGCCTTGGCTGAAGTCACCTTTTGGTGAGCATTTACATGG

GACACAAATATCTTAACATCCTTTGCCCATTTGGAAAGATCTATCCACATACTTCTTCCC

CAGATGTCTTTCTCACCAATTTTCCAATTGTGATCTTTCCAAGTCCCTGACCATCCAGCC

AATCCATTGGCTACAGCCCATGAGTCAATGAATAATCATACATCTGGCCATTTCTTCTTG

CAAACAAACTGTAATACCATGTGTGCTGCCCGAAGTTCTGCCCACTATGAAGATTTCCCT

TCACCTGTGTTTTTCAGGGTTGTCCCAAAAAGGGGTTGTAATGCTGCAGCTGTCCACTTC

TGGGTGGTGCCTGCATAATGTGCAGAGCCATCAGTAAACCAGGCCCTAATCTTCTCCTCT

TCGGTCAGTCGATCATAGGGAACACCCCATGAGGCTATAGGCACATGCTTGGCAGCAGAT

GGCATTGTAACAGGAGTAAAAACCATAGGCATTTGAACAACTTCTTCATGTAACTTGCTT

GTGCCTTCAGGACCTGCTCTGGCCCAATCACATATATACCACTTCCATTTGATAATAGAC

TGCTGCTGTGCACGTCCCACTTTATGACTTGCAGGGTCTAATAGTACCCAGCTCATGATG

GGTAGTTCAGGTCTCATAGTAACTTGGTGTCCTATTGTCAAACGTTCAGTTTCCACTAAG

GCCTAATAGCAGGCCAAGAGCTGTTTTTCAAAGGGAGAATAGTTGTCTGCAGATGATGGT

AGAGCTTTGCTCCAAAATCCCAAAGGCCTTTTCTGTGATTCACCTACAGGGGCCTGCCAG

AGGCTCCAAACAGCATCTCTATCAGCCACAGACACCTCAAGTACCATCGTGTCTGCTAGG

TCATATGGTCCAAGTGGTAGAGCAGCCTGCACAGCAGCCTGGACCTGTTGAAGGGCCTTC

TCCTGTTCCAGGCCCCACACAAAGCTAGCAGCTTTCTTAGTAACTTGGTAAATTCTTGCG

TGTGTTCGCAACCGGCCAGGAAAGACGCAACAAGCCAGAATCTTCTGCGGCAAAGCTTTA

TTGCTTACTCAGGAGCAAGAGAGGAAGAGTGTGGCAGAGAGAGGCAGAGAGGAAGAGAGA

GAGAGGCAGAGAGAGGGGCAGAGAGGGAGAGAGGCAGAGAGCAGAGGAGGAGAGCAGAGG

AAAAGACGAAGAGAAAGAGACGAAGAGAGGGAGTGCCAGAACCCCGTCCCTTTTAAGGAG

AATTATCCTCTGCCTAGGATGTGTCACTCCCTGATTGGCTGCAGCCCATCGGCCCAGTTG

TCATCACGAGAAAGGCAAAGCACATGGCGGGAAAACTGCCCCTGCACATGTGCAGATTAT

TTACTACTTAGAACACAGCTGTCAGCGCCATCTTGTAATGGCAAATGTGAGTGCGGCTCC

CCACAGTAAATAGGCCTAAGTAACACACCCAAGTGAGGGATGTGCTGTCTCCAGAATCCA

AATAGACCCACTAAATGTTGTGCTTCTTTCTTGGTTGTAGGAGGGGCCAGGTGCAATAAC

TTATCTTTCACCTTAGAAGGAATATCTCTACATGCCCCACACCACTGGACTCCTAAGAAT

TTCACTGAGGTAGATGGTCCTTGAATTTTGGTTGGATTTATTTCCCATCCTCTGATATGC

ATATGTGTTACCAATGAGTCCAAAGTGATTGTTACTTCCTGCTCACTTGGTCCAATCAGC

ATAATGTCATCAATATAGTGCACCAATGTGATATTTTGTGGAAGAGCCAAACGATCAAGA

TCCCTTCTAACTAAATTATGGCACAGGGCAGGAGAGTTAATATATCTTTGAGGCAAAACT

GTGAAGGTATACTGTTGGCCTTGCCAACTGAAAGCAAATTGCTTCTGGTGGTCCTTATGG

ACAGGTACTGAGAAGAAGGCATTTGCCAGATCAATAGCTGCATACTAGGTGCTAGGAAAT

GTGTTAATTTGCTCAAGTAATGAAACTACATCTGGTACAGCAGCTGCAATTGGAGTTACT

ACCTGATTTAGTTTTCGATAATCAACTGTCATTCTCCATGATCCATCTGTTTTCTGCACT

GGCCAGATAGGAGAGTTAAAGGGAGATGTATTGGGAACTACCACCCCTGCATCTTTCAAG

TCCTTGATAGTGGCAGTAATTTTTGCAATGCCTCCAGGAATACTATACTGTTTTTGATTC

ACTATTTTCTTTGGCAGAGGCAACTCTGAAGGCTTCCATTTAGCCTTTCCAACCATACTA

GCCCTCACTCTACAGTTCAGGAAACCAATATGAGAATTCTGCCAATTTCTGAGTATATCT

ATCCCAATTATACATTCTGAAACTGGGGAAATGACCGCAGGATGTGTTTGGGGACCTACT

GGACCTACTGTGAGTCGGACATCAGTCAAAACTCCATTAATCACCTGCCCTCCATAAGCC

CCTACTTAACTGGAGGGCCGCAATGTTTCTTGGGATCTCCTGGGATCAGTGTCAACTCAG

AACCAGTATCCAGCAGACCCTGAAAAGTCTGATTATTTCCTTTTCCCCAGTGTACAGTTA

CCCTTGTAAAAGGCTGTAGGTCCCTCTGGGGAAGAACTGGAGAAAGGGTAACAGCAAAAC

CTTTGGGTGTCTTATCAAGATCCTTCCTCAGCGGAACCTGGCCACCCCTTCATTCAAGGG

GTTCCGGATCTGCAAACTGGCTCAAATCTGGAAATTGATTCACTGGCCAAGATTGCCGTT

CACCACGAGCTAATGTGAGAAATTTTCTGCTTATACAGATCAAACAAATATGCAGTAGGC

TTCCTATGTATTTTGTTCCTGGAAACACCATGATTGATTAGCCAATACCAAAGGTCCAAA

CGAGTCATGCCACTATAAATTTCACCTCTCCTGTGCTGACCATTACTGGGTATGTTATTA

TAAACATTGTTTCATCTACGCTGTCCATTATAATAACTAGGATCACCCTGTCTCTGGCGA

TTCAATGCTGTCACCTGGCCCTTGTTACCTCGGAATCCAATTAAACCCATTGAATTTAAT

TCATCTAATTGAGCAGCAGCATCTCCAATGCTAAGGTCTGGCACAGGAAAAGGGAAAGAA

CAAAACCCTTCAAATGTGCTGGTGCCCCTCTCACCAGTTTGTCTTATAGAGCTGATAAAG

GGCATATCTTCTGGACCTTCCCATTGTTGAAGATTAGGTTTTACACAACATATCCACTCT

AGCATTGCAATTTCCCTAAGTCTTAAAATCCCTTCATCAACACTAAGCCACGGAATATTA

GGCATCTCCAAGTCATTTCCAGTAGGCCATCTTTTGATAAACACTTCAGCCAACCATTCA

AACAAACTTTTGACACCTTTTTTTAACGATGCGAGCTTCCGTATTAAACCTAGAATCTCC

ACTCAGAGGGCCCATGTCAGTAAACTCAGCCTGCTCTAGTTTTATGTTCCTACCACCCTA

TCCCACACCCTTAAAATCTATTCCCACACATATTCGCCAGGTTTCTGCTTGAATGAATTA

GCAAACTCATTAAGCTCCTTAGTAGTGTAGCGAATTTCCTCATGGACTACACTTTCTATC

TCCCCTCTAGGAGCCTGTTTTGTTTTGAGTCTGGTTACAGGTCTAGAAGAAACTATTGGT

GGACCTTGAGAAACATCAGTATTGTCTTGTCTGGCATTTTCTTCCGTGAAAGTCAATGCT

GGTTTATCAGACTCTGCAGAATTAATTTCCTCATGTTGGGAAGGCATATTTCAAGGGGTG

GGGCTGAGGGTACTACTTCCTCAGGTGGGGCAAACCCTTGAGAATCTGAAGATTCAAAAT

TCTCAGCTTCAACATGGTCTTCCCACACATCCCCGTCCCAAGTTATAAGATCTCATTCTT

TGCCAATTAGTGCCCTTACTTTAACTGTCGACACACTCTGAGGCTGAGACTTGAATTTTC

GCTGTAGTTCAGCCAACGTTACAATGAGGGTTTCAGATTGATTTTCTGCAACTTGAGCTC

TATGGCTGCAGGAAAGAAGATTCTCTTCAAGGACACACTTAGCAACTTTTAGATCGTTTA

CTTGTGTCTGGAGCTTTTCGATTTTATCACACAACTCTTTCCTTTCATTCATCATTTTTT

CCACAGATACTAAGAGCAGCCAGCCAGTAAAATCATTTTTTGATTTTTCCCCCATCTTGT

AGAAAGCTTTGTACAGTGAATCACCTAATTCATCAAGAAAATCAAGGGCATTAGCTTCTT

TAAGTTTGAAATATAGTTTCAACCATGGGTCTTCAAAATTCTCTGAGCTCCCAGAAGGGG

GAGAATCTGGAGAGGTTTCAGTAGTTGAAAGTGCTGGTGGACCAACAAACCAATCCCAGT

ATTTTAAAAGATTCATCCTTGTACTTCTCTTCCTCTAGAACCACTCCCGGTACCAACTTC

TGTATTAGTCAGGGTTCTCTAGAGTCACAGAACTTATGGATAGTCTCTAGATAGTAAAGG

AATTTATTGATGACTTACAGTCTGCAGCCCAATTCCCAACAATGGTTCAGTAGTAGCTGT

GAATGGAAGTCCAAAGATTTAGCAGTTACTCAGTCTCACACAGCAAGCAGGCGAAGGACA

AGAGCAAGACTCCCTTCTTCCAATGTCCTTATACTGTCTCCAGCAGAAGGTGTAGGCCAG

ATTAAAGGTGTGAACACACCTTTAATCTGGGATGACCTTGAACTCAGAGATTTAGTCTTC

TGGAATCCATAGCCACTCTGCCTCGAGATCTCCATACCAAGATCCAGATCAGAAACTTCT

ATCTCCAAGCCTCCAGATTCCGGATTGTAGTCCATTCCAAATATAGTCAAGTTGACAACC

AGGAATAGCCACTACAGTATGATTTTTTTGTTCCACTTTTATATTAACTTCAATTCTTTG

CAACATTCGTTAACTTTGAAGTGTGAATCCACCAAGGCTGTACTTTTGCTACAAAGAACA

GTAGTCTTTTGTTCTTTCTGGTTTAGATGTTCCATTTGTAACTATTTGTAACTAGTGACT

GGGGACTCTCCACAACACACACAGAGGGCATATATGTGTGTGTGTGTGTGTGTGTGTGTA

TACATACACATATATATACACCACACAAATATATGTATATATATTTATGTGTATATATAT

ATATATGTGTGTGTGTAATACATGCGTACACATATAATAGAGTTGTTTATGTACAGGTTC

TGTATGGGGTAACGTCTTATCTCTCGTGTGTGTGTGTGTGTGTGTGTGTGTGTGTGTGTG

TGTGTGTGTGTGTGTGTGTGTGTGTGTGCACATGCTTGTAGAGATAGAGATCAACTTTGG

GTGTTGTTCTTTAGGAGCCATTCACCTTGTATTTGAAACAGGGTCTCTCTTTGGGGCCTG

TGAATTACCTATCAGGATAAACTGGGTGGACAGTGAGCTCCAAGAGTGTTCTTGTTTCCA

TCTCCACAAGGCTGAGATTACAAGCAAGTTAGCATGCTTGACTTTCTTTAAGAAAAAAAA

AAAGGTTATGGAAATACAATGTATATTCTCATACTTGCATGGCAAAAACTTACCTGACTG

AGCTCTCTCCACAGTCCCAGCAGTTGCATGTATTTACATCTATAAACAAAGAACTTGTGA

ACCTGTTTCCCAGAACCACTGTAAACAAACAATTTGTGAACATGTTTCCCAGAACTAGAG

TCTGAGCCATCTATCTCCATAGGAAAAAAAAAAAAAACAATCTGAACCAAAACACAACAA

AAAACAACTTATGTTAGCATTTAGTCCACCATCAGCCACTTTTCTGGGAAGTTGCTAATC

AAGAAGAATACATTGATATTTCTGTAATTCGCTGCCCCTAACCTTTAGTGCACAAAGCAG

CTTTGTTTCAGGCATTAATTCAACATATCATTGTCTTAAGATGGAAAGAATGGTCAAGAA

TGCAGATTTTTTTGTTGTTGTTGTTGCTGGGTTTTTTGTTTTGTTTTGTTTTGTTTTGTT

TTGTTTTGTTTTGTTTTGTTTTGTTTTTTGAGACAGGGTTTCTCTGTATAGCCCTGGCTG

TCCTGGAACTCACTCTGTAGACCAGGCTGGCCTCCAAATCAGAAATCTGCCTACCTCTGC

CTCCCAAGTGCTGGGATTAAAGGCGTGCACCACCACGCCCAGCCCAAGAATGCAGAGTTT

GATTGCTTGATTTTGTGTAATTTTGCAGAAAAAGAGAAGAAATCAATAAGTAGAATAGAT

TGGCCTCATACTAGACACTAAAAAGATACCTAAGAAAAGACATTAGCCTCCAAGTCATCA

AAAAGCATCCCCGGAAAATGGATGTATTGCTTTACTACAACAAATCAGTCATATTGGCCT

TAAAATTGTGGACAAATGTCTTTCATGGGTGACTGGCACACCTTCCTCACTTAACATATT

ACTCTTTATTACCTCCCATTTTTAACGTGTGAAGAATCAATTTACAGCCATTTCTTCTGA

GAATCTTTGATCCAGATGATTTGTCTATATGCATTGTTATAGTACATGTGATACTCTTAT

TATCTAGGTGTCTCACCATGGCCCTCATCTCCAGGCTTCTCATCTGATGACCTGATATTG

TTGCTTTGCTGGGCATGTTTGATGAATGACAATAAAAAAAAGTGTGTTGAAATACTCCTT

TTCGAAATTTCCTTATGAGATAGAAACTTTCTATTCAAGTTCCATGCATTGTCCAACTCC

CCTCCCATCTCACCTCTCTGTGTAGGAAACCCATTCCTCTGTAATGAAGTTTACCCACCA

CCTGTTCCCTGACTCATGTTATTCACAGTAACACATACACTCTCTGTTTATTTTCCTGAC

TACATTGAATTTTGCTGGTTTTGTTCATTTGCTCTGTGGTGACTGAACCTAGGGCCTATC

CTATGATAGTGAAGAGCTTCACCACAGTTCTGTATTTCTAGTCCTGGCATGATATTTCTT

GAAGGCATTTTAACCACAGAGCCAACAATAATGCTGAATCCAACATATTTACAGTAAAGC

CTGGTTAGATAAATATCCTGCATTCCACAACTCTAGCTACTTTATATATAAGCACCTGAG

TGGCTTCTGTAAGTGTTTTTGTAGTTGTTGTTGTTTGTTTGTTTTATTAACTTGCTATTT

TTTTAAATCTGAGATTGTGGATTTCCTAATGAAAGAATAAAATCTCATTTTCATTTTTTT

TCAGGTCCTCGGCCCAGGTGTTGGCATCCTTTCTGTATGCATTGGAGCAGAGATTGGACT

TTCTATGGCTATTAACCTAAAACAAATAAGAGCCACTGTACTTATCAATGGGCCTAATTT

TGTTTCTCAAAGTCCACATGTATATCATGGTCAGGTCTACCCACCTGTACCCAGTAATGA

AGAGTTTGTAGTCACCAATGCCTTGGGACTTGTAGAATTCTATCGAACCTTTCAGGAAAC

TGCAGATAAGGACAGCAAATATTGTTTTCCCATTGAAAAAGCTCATGGACATTTCCTTTT

TGTGGTTGGAGAAGATGATAAAAATCTCAACAGCAAAGTGCATGCTAATCAAGCCATAGC

ACAGCTGATGAAAAATGGAAAGAAGAATTGGACTCTGCTGTCTTACCCTGGGGCAGGTCA

CCTGATTGAGCCTCCCTATACCCCACTGTGCCAAGCCTCAAGGATGCCCATTTTGATCCC

AAGCCTCAGCTGGGGAGGAGAGGTTATCCCCCATGCAGCTGCACAGGAGCATTCTTGGAA

GGAGATACAGAAATTTCTCAAGCAGCATCTCCTTCCAGATTTGAGCAGTCAGCTCTGAGT

GGACTTGATTATATTCCTGGAAAGTGGAGCTGGGCATCTCCTGGCCAGCACCACTCCTCA

CTTCCATAGAGGAATGTCTTTGATCTCTTATCTGGCAAGGAAGGAGAGTACCACAAGAAA

ATACAGGAGGATGGAGAGTGATAACGTCTTGAATTTGGAAGGGGAAACATGTTTTCCATG

GAATGAAATGTCATGCAGTGAGAGCCCTATATCTACATGAATAAAATCGTAGGCCTTTCC

TAAAATGTTCAACATCATAGCAACTTTCTGTTATGATAATTATCAGGGAAATTATCAGTG

ATAAACCACAGAATACTTTTGTTTATAAAAGAAACATGAAAATAATTATATATTATCACT

TATTAATTTCTTGAAACTCACATTAAATATACTTAG

Size bp

100

300

700

500

1500

3000

1000
